# The germline coordinates mitokine signaling

**DOI:** 10.1016/j.cell.2024.06.010

**Published:** 2024-07-02

**Authors:** Koning Shen, Jenni Durieux, Cesar G. Mena, Brant M. Webster, C. Kimberly Tsui, Hanlin Zhang, Larry Joe, Kristen M. Berendzen, Andrew Dillin

**Affiliations:** 1 Department of Molecular & Cellular Biology, University of California, Berkeley, Berkeley, CA, 94720, USA; 2 Howard Hughes Medical Institute, University of California, Berkeley, Berkeley, CA, 94720, USA.; 3 Present affiliation: Stanford University, Palo Alto, CA, USA.; 4 Weill Institute for Neurosciences, University of California, San Francisco, San Francisco, CA, USA.; 5 The Helen Wills Neuroscience Institute, University of California, Berkeley, Berkeley, CA, 94720, USA.; 6 Lead contact

## Abstract

The ability of mitochondria to coordinate stress responses across tissues is critical for health. In *C. elegans*, neurons experiencing mitochondrial stress elicit an inter-tissue signaling pathway through the release of mitokine signals, such as serotonin or the Wnt ligand EGL-20, which activate the mitochondrial unfolded protein response (UPR^MT^) in the periphery to promote organismal health and lifespan. We find that germline mitochondria play a surprising role in neuron-to-periphery UPR^MT^ signaling. Specifically, we find that germline mitochondria signal downstream of neuronal mitokines, Wnt and serotonin, and upstream of lipid metabolic pathways in the periphery to regulate UPR^MT^ activation. We also find that the germline tissue itself is essential for UPR^MT^ signaling. We propose that the germline has a central signaling role in coordinating mitochondrial stress responses across tissues, and germline mitochondria play a defining role in this coordination because of their inherent roles in germline integrity and inter-tissue signaling.

## Introduction

Multi-cellular organisms must coordinate the health and function of tissues across the organism to maintain health. This requires specialized tissues or organs that can communicate long-range signaling with other tissues to ensure organismal health. For instance, the central nervous system regulates the activity of other organs through hormones or neuronal signaling (e.g. neurotransmitters and neuropeptides). These inter-tissue signaling pathways are especially crucial during conditions of stress or during unhealthy aging, where aging-induced dysfunction in certain organs may contribute to loss of inter-tissue communication pathways and a broader loss of homeostasis^1,2^.

Mitochondria are a prominent source for these inter-tissue stress signals^3–5^. This is perhaps due to their endosymbiotic origin: When the prokaryotic mitochondrial precursor was engulfed by its now-eukaryotic host, mitochondria had to develop signaling pathways to communicate their needs with the new host cell and nuclear genome. Fittingly, mitochondria have developed stress signaling pathways that communicate stress between different tissues to activate protective cellular programs, and these pathways are highly conserved from invertebrates through mammalian species. One such pathway is the mitochondrial unfolded protein response (UPR^MT^), a transcriptional program activated upon mitochondrial stress to alleviate mitochondrial burden and promote cellular and organismal health^6–8^. In mice, low-level mitochondrial stress in POMC neurons signals to white adipose tissue to activate the UPR^MT^ to protect against obesity and enhance metabolic turnover^9^. In *Drosophila*, mild mitochondrial stress in muscle tissue activates the UPR^MT^ to promote overall longevity and muscle function^10^.

**Figure 1:**
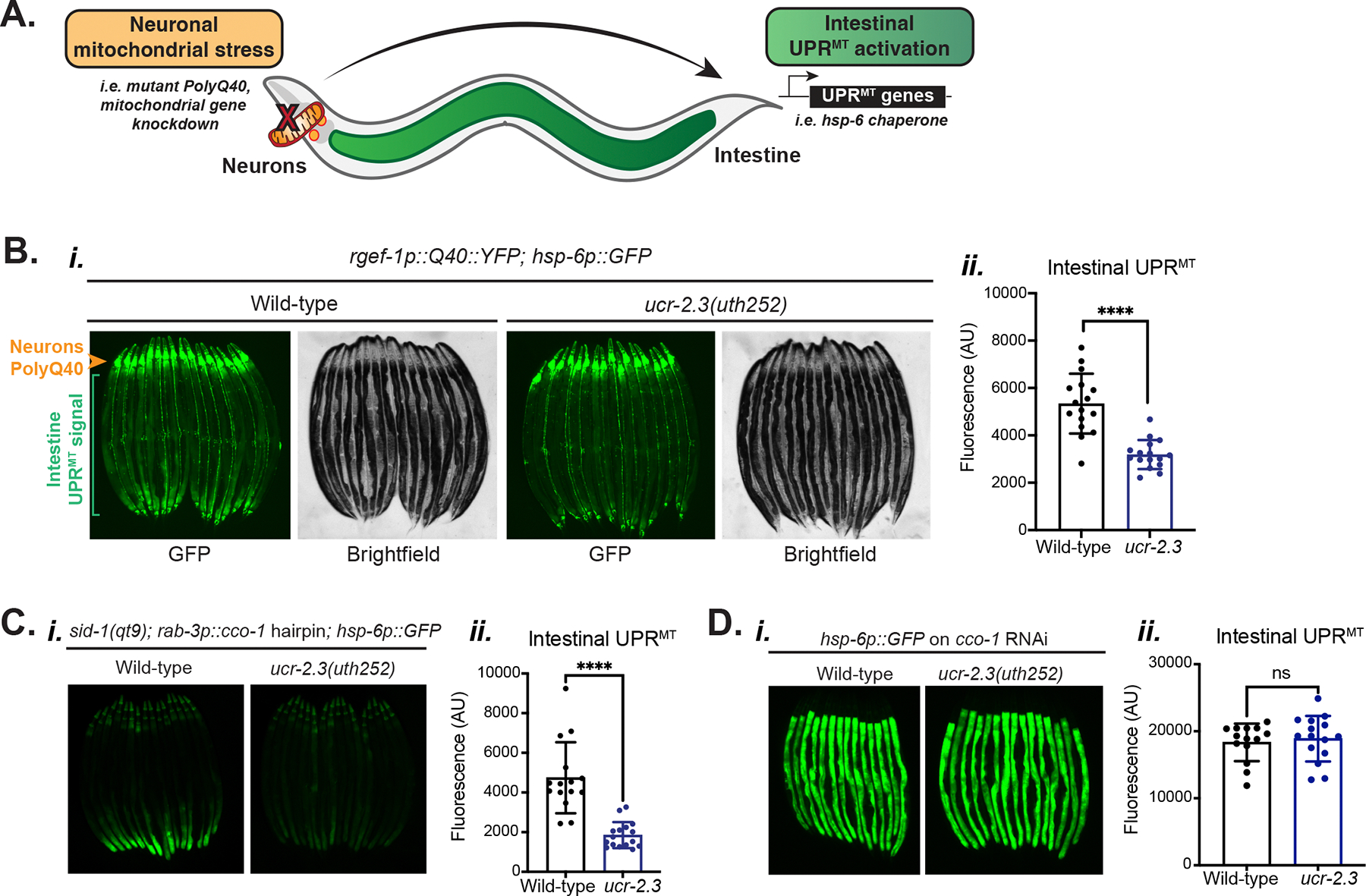
Mutagenesis screen reveals the requirement of *ucr-2.3* in neuron-to-intestine UPR^MT^ signaling a. Schematic of cell non-autonomous UPR^MT^ signaling model in *C. elegans*. b. Fluorescence imaging *(i)* and quantification *(ii)* of intestinal UPR^MT^. The *Q40::YFP* signal in neurons is denoted with an orange arrowhead. The intestinal UPR^MT^ transcriptional reporter signal *hsp-6p::GFP* is denoted with green brackets. ****p<0.0001; n > 3. c. Fluorescence imaging *(i)* and quantification *(ii)* of intestinal UPR^MT^ ****p<0.0001; n > 3. d. Fluorescence imaging *(i)* and quantification *(ii)* of intestinal UPR^MT^. p = 0.6372; n > 3. See also Figure S1 and Figure S2.

Much of the work investigating the mechanisms of inter-tissue mitochondrial signaling has been performed in *C. elegans* using a model of inter-tissue (or cell non-autonomous) UPR^MT^ communication between neurons and the intestine^11^. In *C. elegans*, neurons experiencing mitochondrial stress, such as neuron-specific knockdown of mitochondrial genes^11^, expression of the aggregation-prone PolyQ40 protein, or neuron-specific targeting of KillerRed to mitochondria^12^, activate the UPR^MT^ in the intestine, a tissue completely peripheral and non-innervated by neurons in *C. elegans*, to protect overall organismal health (Fig 1A). So far, several neuronal factors that mediate neuron-to-intestine UPR^MT^ signaling in *C. elegans* have been identified. Neuron-specific over-expression of the histone demethylases *jmjd-1.2* and *jmjd-3.1* activate intestinal UPR^MT^ in *C. elegans* and regulate UPR^MT^ activation in mice^13^. Neurons experiencing mitochondrial stress release neurotransmitters such as serotonin or the neuropeptides FLP-1 and FLP-2 or the Wnt ligand, EGL-20, to activate intestinal UPR^MT^ in *C. elegans*^12,14–16^.

The *C. elegans* Wnt ligand EGL-20 has garnered particular interest as it was identified as a bona fide mitochondrial signaling molecule, or “mitokine”, because it is both necessary and sufficient for cell non-autonomous activation of the intestinal UPR^MT 16^. However, neuronal release of Wnt/EGL-20 in this cell non-autonomous context does not induce the canonical Wnt developmental program in the intestine. Therefore, we reasoned that there must be additional regulatory mechanisms for cell non-autonomous activation of the UPR^MT^ in the periphery. To answer this, we performed a genome-wide mutagenesis screen to identify additional mechanisms that could mediate UPR^MT^ cell non-autonomous signaling between neurons and the intestine in *C. elegans*. Through this screen, we identified *ucr-2.3* as essential in mediating cell non-autonomous UPR^MT^. Interestingly, *ucr-2.3* is a mitochondrial gene that is expressed primarily in a few neurons and in the germline. We discovered that *ucr-2.3* activity in the germline mediates UPR^MT^ signaling between the neurons and intestine. We further found that the germline and germline mitochondria play a general role in mediating cell non-autonomous UPR^MT^ signaling through regulating lipid metabolism and transport pathways between reproductive and intestinal tissues. These data indicate that integrity of the germline tissue, mediated through germline *ucr-2.3* mitochondrial activity, is crucial for neuron-to-intestinal UPR^MT^ signaling. Overall, our work identifies a signaling role for the germline, once thought of as a tissue insulated from the soma, in directly communicating with somatic tissues to regulate organismal stress responses and metabolism. Our results provide broader implications for the importance of germline mitochondrial quality, its interactions with neuronal signaling and intestinal lipid metabolism, and the consequences of these interactions on organismal health.

## Results

### Mutagenesis screen reveals the requirement of *ucr-2.3* in neuronal UPR^MT^ signaling

To identify additional regulators of inter-tissue (or cell non-autonomous) mitochondrial signaling, we conducted a genome-wide mutagenesis screen using a genetic model in *C. elegans* where neurons expressing an aggregation-prone mutant PolyQ40 protein fused to YFP (*rgef-1p::Q40::YFP*) activates the UPR^MT^ in the intestine^12^, which we assessed using a GFP transcriptional reporter for expression of *hsp-6*, the mitochondrial Hsp70 chaperone induced by the UPR^MT^ (*hsp-6p::GFP*) ^8^ (Fig 1A). After identifying mutants with suppressed intestinal UPR^MT^, but intact neuronal PolyQ40 signal, we used single nucleotide polymorphism (SNP) mapping and whole genome sequencing approaches^17^ to identify a missense mutation in the gene *ucr-2.3* as a causal mutation for suppressed cell non-autonomous UPR^MT^ (Supp Fig 1A, F). We verified the necessity of *ucr-2.3* in cell non-autonomous UPR^MT^ signaling by creating several alleles of early stop codon, nonsense-mediated decay (NMD) mutations in *ucr-2.3* using CRISPR/Cas9 genome editing and observed that they all suppressed PolyQ40-mediated cell non-autonomous UPR^MT^ (Fig 1B; Supp Fig 1B, F). The early stop codon mutation in the *ucr-2.3(uth252)* allele leads to almost complete loss of gene expression, effectively creating a null allele (Supp Fig 1D, F). We also tested another *ucr-2.3* mutant containing a large deletion resulting in an early stop codon and found that it also suppressed cell non-autonomous UPR^MT^ (Supp Fig 1C, F). We further tested whether *ucr-2.3* was involved in other models of cell non-autonomous UPR^MT^ signaling, such as the neuron-specific knockdown of the mitochondrial complex IV subunit *cco-1* (*rab-3p::cco-1* hairpin in the *sid-1(qt9)* genetic background)^11^ and found that the *ucr-2.3* null mutation also suppressed this model of cell non-autonomous UPR^MT^ (Fig 1C). We concluded that *ucr-2.3* is as a key regulator of neuron-to-intestine UPR^MT^ signaling induced by multiple forms of neuronal mitochondrial stress.

We next asked whether *ucr-2.3* was required for cell autonomous induction of the UPR^MT^. We tested this by feeding *cco-1* RNAi, which targets a mitochondrial complex IV subunit, to wild-type and *ucr-2.3* mutant strains in the *hsp-6p::GFP* UPR^MT^ reporter background with and without neuronal PolyQ40 expression and found that *ucr-2.3* mutant animals still activate, not inhibit, the UPR^MT^ cell autonomously (Fig 1D, Supp Fig 1E). These data indicate that *ucr-2.3* is not involved in the general machinery of UPR^MT^ activation, but rather has a specific role in cell non-autonomous signaling of mitochondrial stress. We also excluded the possibility that loss of *ucr-2.3* leads to a temporal delay in non-autonomous UPR^MT^ signaling, since the suppression of non-autonomous UPR^MT^ signaling by the *ucr-2.3* mutation increases over aging (Supp Fig 1G). Finally, we tested the specificity of *ucr-2.3* loss in non-autonomous stress signaling and found that *ucr-2.3* loss does not suppress cell non-autonomous signaling of the UPR^ER^ or the cytosolic heat stress response (HSR) between neurons and the intestine (Supp Fig 2A–B). Conversely, there is a slight up-regulation of both stress responses upon *ucr-2.3* loss. Overall, our data indicate that *ucr-2.3* is a critical and specific gene required for mediating cell non-autonomous UPR^MT^ signaling between neurons and the intestine in *C. elegans*.

### UCR-2.3 is a mitochondrial protein with a non-canonical role in mitochondrial stress signaling

The *ucr-2.3* gene encodes UCR-2.3 in *C. elegans*, which is reported to be a mitochondrial protein homologous to mammalian UQCRC2, a core subunit of respiratory complex III of the electron transport chain (ETC)^18,19^. That the loss of *ucr-2.3* results in a suppression of UPR^MT^ signaling was perplexing because the knockdown of many mitochondrial proteins, especially those involved in the ETC, activate, not suppress, the UPR^MT 6,11,20^. Indeed, we observed that even in “basal” conditions, i.e. no additional source of stress, the *ucr-2.3* null mutation does not activate intestinal UPR^MT^ (Supp Fig 2C), suggesting that UCR-2.3 may have a non-canonical mitochondrial role in regulating UPR^MT^ signaling.

**Figure 2:**
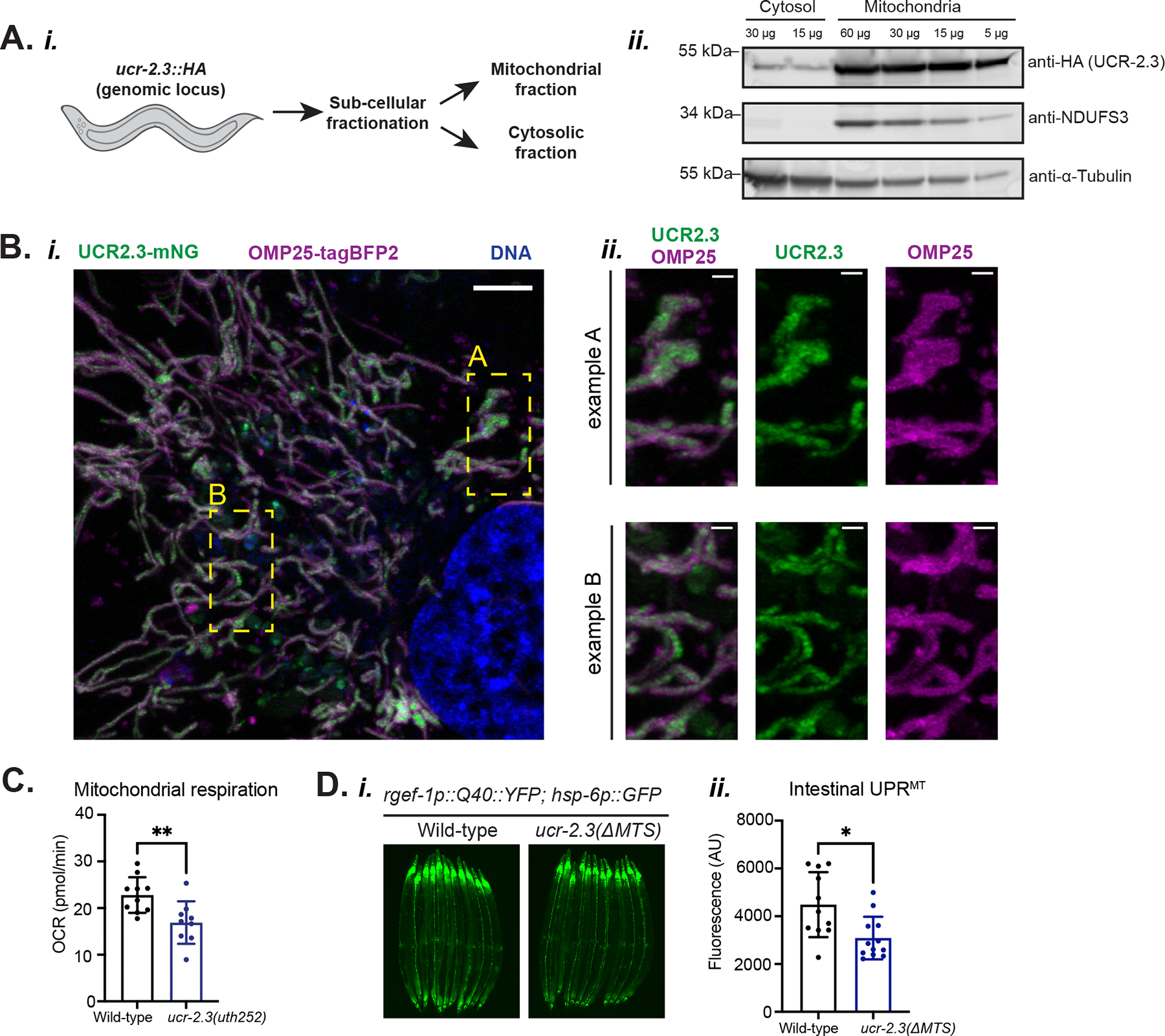
UCR-2.3 is a mitochondrial protein in *C. elegans* with a non-canonical role in cell non-autonomous UPR^MT^ signaling a. *(i)* Sub-cellular fractionation schematic. *(ii)* Cytosolic and mitochondrial fractions run on a western blot probed with antibodies to detect HA-tag (for the UCR-2.3 protein), NDUFS3 (a mitochondrial protein), and a-tubulin (a cytosolic protein). b. *(i)* Live-cell imaging showing sub-cellular localization of *C. elegans* UCR-2.3 in human RPE1 hTert cells. Scale bar = 5 μm. *(ii)* Close up of two example regions shown in *(i)*. Scale bar = 1 μm. Data shown is representative of at least two independent imaging experiments with 5–10 images collected per cell line. c. Measurement of mitochondrial respiration (OCR, oxygen consumption rate). **p = 0.0079; n > 4. d. Fluorescence imaging comparison *(i)* and quantification *(ii)* of intestinal UPR^MT^ between wild-type and a *ucr-2.3* mutant with a deletion of the predicted mitochondrial targeting sequence (MTS). *p = 0.0103; n = 3. See also Figure S2.

We confirmed that UCR-2.3 is a mitochondrial protein using three orthogonal approaches. First, when we sub-cellular fractionated *C. elegans* lysate with an HA-epitope inserted at the *ucr-2.3* genomic locus using CRISPR/Cas9 editing, we found that the UCR-2.3 protein was enriched in the mitochondrial fraction, similar to that of NDUFS3, a subunit of Complex I, and as opposed to a-tubulin, which was detected predominantly in the cytosolic fraction (Fig 2A). Second, using human RPE-1 cells, we found that over-expression of the *C. elegans* UCR-2.3 protein tagged with mNeonGreen thoroughly co-localized with an outer mitochondrial membrane protein OMP25 tagged with BFP (mean 71.8%, SD = 18.2%) (Fig 2Bi). Indeed, we observed that UCR-2.3 primarily localizes within the OMP25 signal (Fig 2Bii), suggesting that UCR-2.3 is located within the mitochondrial matrix, consistent with its homology to UQCRC2, the matrix-facing core subunit of complex III in the mitochondrial ETC^18^. Third, we observed that UCR-2.3 functionally acted as a mitochondrial ETC protein as the *C. elegans ucr-2.3* mutant had decreased mitochondrial respiration (Fig 2C). Thus, we conclude that *ucr-2.3* encodes a bona fide mitochondrial protein in *C. elegans*, which has a similar sub-cellular localization and function to its mammalian ortholog UQCRC2.

Next, we tested whether UCR-2.3 mitochondrial localization was essential for its role in mediating cell non-autonomous UPR^MT^ signaling. Using CRISPR/Cas9 genomic editing, we excised the predicted mitochondrial targeting sequence (MTS)^21^ from UCR-2.3 and found that this homozygous mutant *ucr-2.3(*Δ*MTS)* suppressed intestinal UPR^MT^ signaling, much like the loss of function mutant (Fig 2D), without affecting *ucr-2.3* expression (Supp Fig 2D). Overall, we concluded that *ucr-2.3* encodes a mitochondrial localized protein whose mitochondrial function is important for regulating neuron-to-intestine cell non-autonomous UPR^MT^ signaling in a manner atypical of canonical mitochondrial ETC proteins.

### *ucr-2.3* has a unique role within the UCR-2 gene family in *C. elegans*

**Figure 3:**
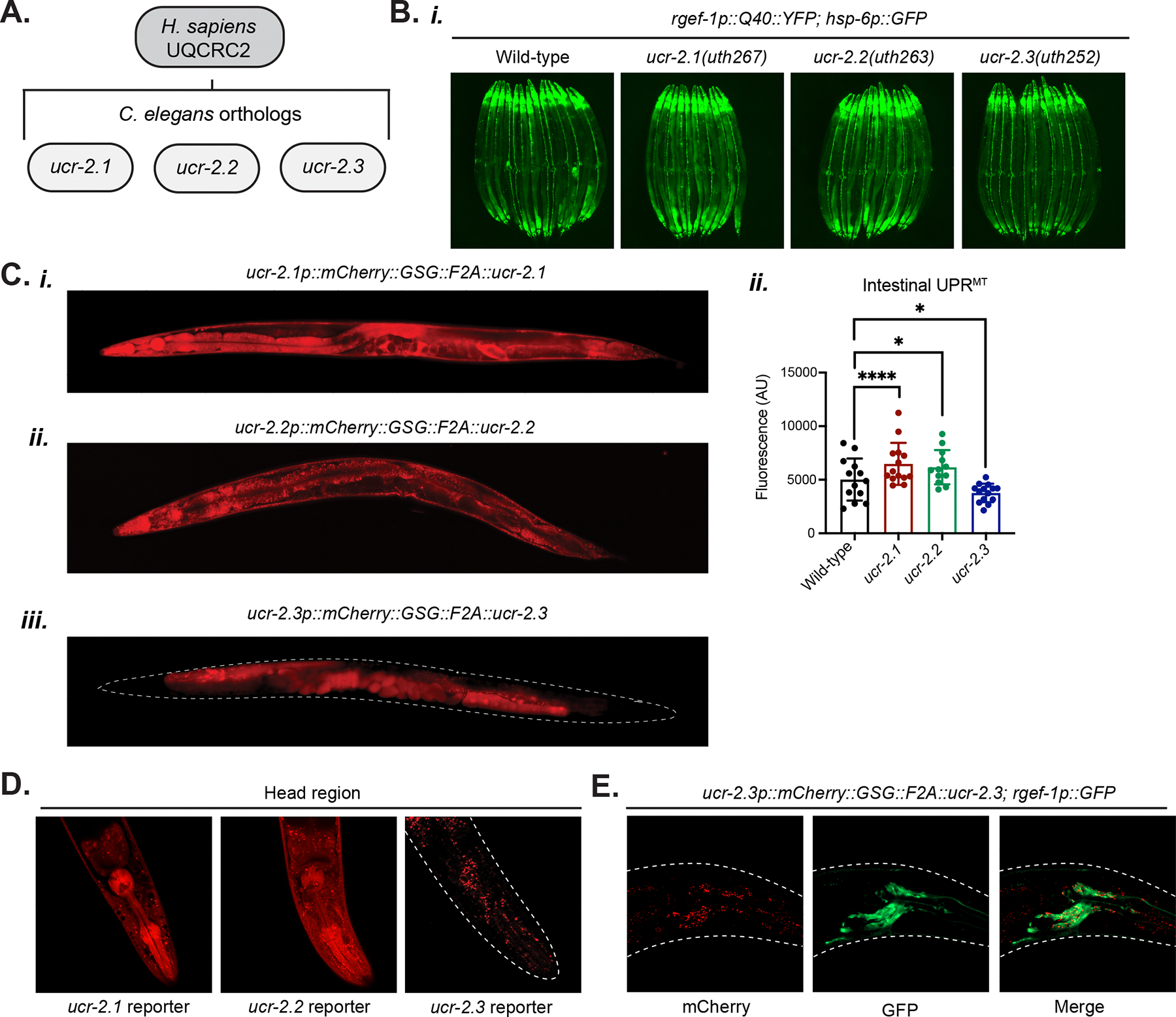
*ucr-2.3* is part of the UCR-2 family of genes that differ in their tissue expression and role in cell non-autonomous UPR^MT^ signaling a. Human UQCRC2 is homologous to a family of UCR-2 genes in *C. elegans* including *ucr-2.1*, *ucr-2.2*, and *ucr-2.3*. b. Fluorescence imaging comparison *(i)* and quantification (*ii*) of intestinal UPR^MT^. *p < 0.05, ****p < 0.0001; n = 3. c. Fluorescence imaging comparison of tissue expression patterns across the UCR-2 family genes using an endogenous transcriptional expression reporter for each gene: *ucr-2.1* (*i*), *ucr-2.2* (*ii*), and *ucr-2.3* (*iii*). d. Close up head images of the endogenous transcriptional reporter as described in (C). e. Close up head imaging of *ucr-2.3* endogenous transcriptional reporter crossed to a *rgef-1p::GFP* reporter strain, expressing GFP in neurons. See also Figure S3.

*ucr-2.3* belongs to a family of paralogs in the UCR-2 family in *C. elegans* that are all homologous to mammalian UQCRC2: *ucr-2.1, ucr-2.2,* and *ucr-2.3* (Fig 3A). To investigate whether the other paralogs of the UCR-2 family were also involved in cell non-autonomous UPR^MT^ signaling, we made similar early stop NMD mutations in *ucr-2.1* and *ucr-2.2* using CRISPR/Cas9 editing (Supp Fig 3Ei–ii). Surprisingly, we found that only loss of *ucr-2.3* suppressed cell non-autonomous UPR^MT^; loss of *ucr-2.1* or *ucr-2.2* slightly activated, not suppressed, UPR^MT^, as expected for the knockdown of canonical mitochondrial genes (Fig 3B). This suggested that *ucr-2.3* has a unique role among the UCR-2 family genes compared to *ucr-2.1* and *ucr-2.2*.

We continued to explore the functional and genetic relationships among the UCR-2 family genes and observed that while *ucr-2.1* and *ucr-2.2* were genetically and functionally redundant with each other, neither were redundant with *ucr-2.3*. For instance, while knockdown of *ucr-2.3* reduced mitochondrial respiration (Fig 2C), individual knockdown of either *ucr-2.1* or *ucr-2.2* had no effect on respiration. Only the double knockdown of *ucr-2.1* and *ucr-2.2* could reduce mitochondrial respiration (Supp Fig 3A). Similarly, while the individual knockdown of *ucr-2.1* and *ucr-2.2* had no effect on the UPR^MT^, their double knockdown strongly activated UPR^MT^ (Supp Fig 3B), again as expected for the knockdown of a canonical mitochondrial ETC gene^6,11^. However, the double knockdown of either *ucr-2.1*/*ucr-2.3* or *ucr-2.2*/*ucr-2.3* could not activate UPR^MT^. Only conditions in which both *ucr-2.1* and *ucr-2.2* are knocked down could activate UPR^MT^ (Supp Fig 3C). Transcriptionally, we found that while knockdown of *ucr-2.3* increased the expression of *ucr-2.1* and *ucr-2.2* (Supp Fig 3D), knockdown of either *ucr-2.1* or *ucr-2.2* individually or together had no effect on *ucr-2.3* expression (Supp Fig 3E). This suggested that while loss of *ucr-2.3* requires compensation from *ucr-2.1* and *ucr-2.2* equally, loss of either *ucr-2.1* or *ucr-2.2* does not require *ucr-2.3*. Altogether, these data suggest that *ucr-2.1* and *ucr-2.2* are functionally redundant with each other for a role in the mitochondrial ETC, likely together recapitulating the role of the mammalian UQCRC2 complex III subunit, while *ucr-2.3* may have a unique genetic and functional role within the UCR-2 family.

Most strikingly, we found that *ucr-2.3* differs from *ucr-2.1* and *ucr-2.2* in its tissue expression pattern (Fig 3C–D). Through searching transcriptomic databases, we found that while expression of *ucr-2.1* and *ucr-2.2* is ubiquitous in most tissues, *ucr-2.3* expression is enriched in the germline and neuronal cells^22–24^ (Supp Fig 3F). We confirmed this by generating an endogenous expression reporter for each of the *ucr-2* genes by inserting an mCherry::F2A construct between the promoters of *ucr-2.1*, *ucr-2.2*, and *ucr-2.3* and the respective CDS at the genomic loci using CRISPR/Cas9 editing (Fig 3C). The expression patterns confirm that *ucr-2.1* and *ucr-2.2* are ubiquitously expressed throughout the somatic tissues, though largely excluded from the germline (Fig 3Ci–ii, 3D). In contrast, *ucr-2.3* expression was exclusively localized to the germline as well as a few cells in the head of the animal (Fig 3Ciii, 3D). We found that expression of *ucr-2.3* in head cells mostly overlapped with GFP signal from a pan-neuronal *rgef-1p::GFP* reporter strain (Fig 3E), suggesting that the cells expressing *ucr-2.3* are indeed neurons. Note that we did observe that the F2A self-cleaved mCherry protein appeared to aggregate in these neurons, which has been previously observed^25,26^. Finally, we also imaged a *ucr-2.3* endogenous translational reporter strain that contained a GFP inserted at the genomic locus of *ucr-2.3* using CRISPR/Cas9 editing and found that the sub-cellular localization of UCR-2.3 was indeed targeted to mitochondria within the germline (Supp Fig 3G), consistent with a previous study^27^. In summary, our data indicate that while *ucr-2.1* and *ucr-2.2* are more ubiquitously expressed throughout the somatic tissues of *C. elegans*, *ucr-2.3* expression is restricted to mitochondria in the germline and certain neuron cells.

### The germline role of *ucr-2.3* is critical for mediating non-autonomous UPR^MT^

**Figure 4:**
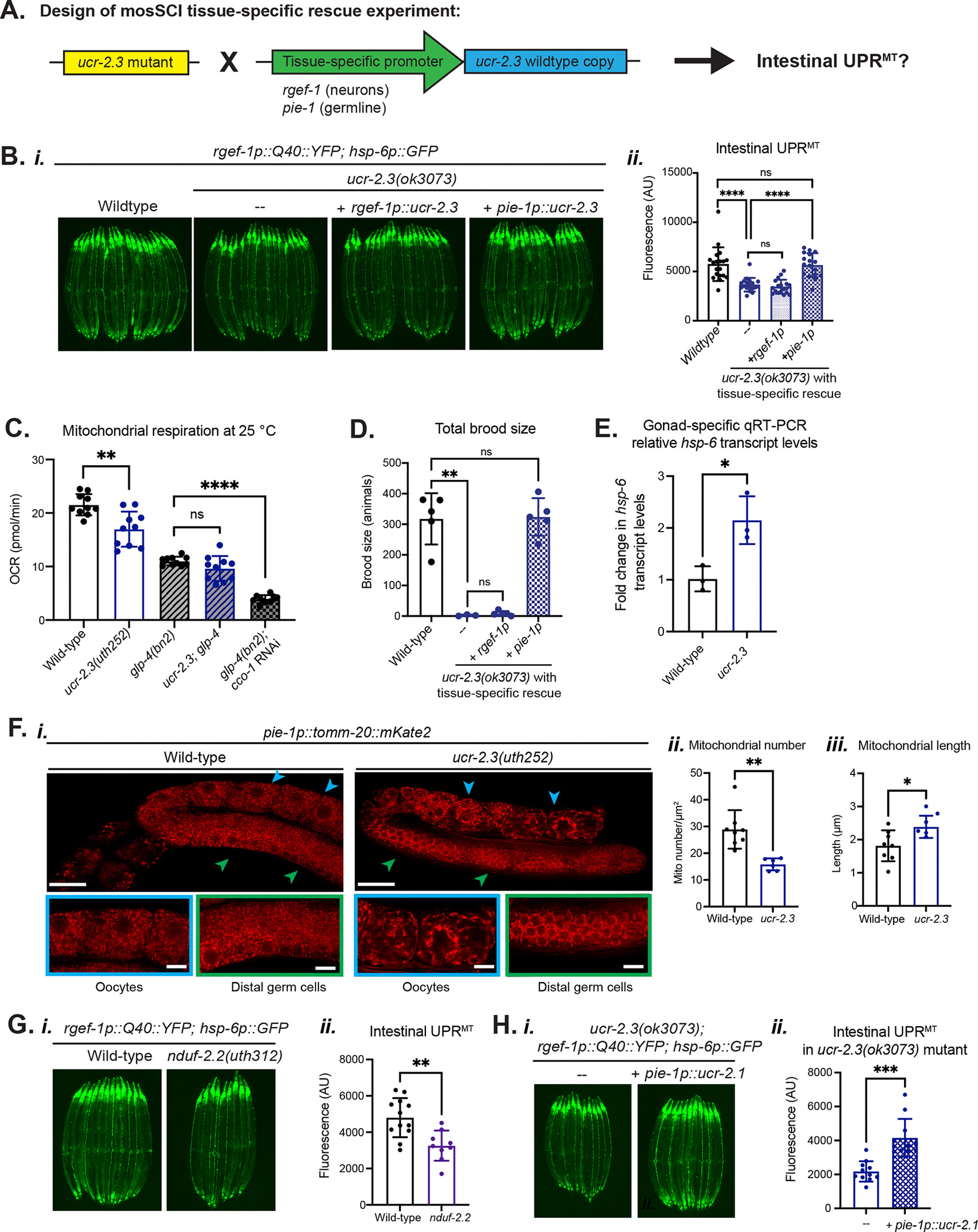
*ucr-2.3* acts in the germline to mediate cell non-autonomous UPR^MT^ signaling and germline mitochondrial integrity a. Single-copy (mosSCI) tissue-specific rescue experiment for intestinal UPR^MT^ signal in the *ucr-2.3* mutant background. b. Fluorescence imaging comparison *(i)* and quantification *(ii)* of intestinal UPR^MT^ in the tissue-specific mosSCI rescue experiment. ****p< 0.0001, all non-significant p values > 0.3924; n = 3. c. Measurement of mitochondrial respiration (OCR) at the restrictive temperature 25°C. All strains were fed control HT115 RNAi bacteria, except for the *glp-4(bn2)* mutant animals fed *cco-1* RNAi. **p = 0.0019, ****p < 0.0001, non-significant p = 0.1051; n = 3. d. Measurement of total brood size. **p=0.0011, all non-significant p values > 0.1507; n = 3. e. qRT-PCR comparison of *hsp-6* transcript levels in gonads. Transcript levels normalized by *rpl-32*. *p = 0.0326; n = 3. f. Fluorescence widefield imaging of germline mitochondria *(i)*. Scale bar = 25 μm. Distal germ cells (green arrowhead) and oocytes (blue arrowhead) are shown in zoomed in images (scale bar = 10 μm). Images shown are representative of at least 5–10 animals imaged per strain in at least three independent experiments each. Quantification of mitochondrial number *(ii)* and mitochondrial length *(iii)*. **p = 0.0010, *p = 0.0205; n > 2. g. Fluorescence imaging comparison *(i)* and quantification *(ii)* of of intestinal UPR^MT^. **p = 0.0015; n > 3. h. Fluorescence imaging comparison *(i)* and quantification *(ii)* of intestinal UPR^MT^. ***p=0.001; n = 2. See also Figure S4 and Table S1.

We next asked whether this tissue-specific expression pattern of *ucr-2.3* in either the neurons or germline conferred its unique ability to mediate cell non-autonomous UPR^MT^ signaling. We designed a tissue-specific rescue experiment where we knocked in an integrated, single-copy (mosSCI^28^) of wild-type *ucr-2.3* driven by a tissue-specific promoter for either neurons (*rgef-1p*) or the germline (*pie-1p*) into the *ucr-2.3* mutant strain and asked whether this neuron or germline-specific rescue of *ucr-2.3* could restore cell non-autonomous UPR^MT^ signaling (Fig 4A). We found that neuron-specific rescue of *ucr-2.3* expression had no ability to restore the suppressed cell non-autonomous UPR^MT^ signal of the *ucr-2.3* mutant (Fig 4B, ***rgef-1p***). However, germline-specific rescue of *ucr-2.3* expression could completely restore the cell non-autonomous UPR^MT^ signal in the *ucr-2.3* mutant (Fig 4B, ***pie-1p***). This indicated that *ucr-2.3* primarily acts in the germline, not neurons, to mediate cell non-autonomous UPR^MT^ signaling.

To further test the tissue-of-action for *ucr-2.3* activity, we also used the FLP/FRT recombinase system to test how a neuron-specific knockout of *ucr-2.3* affected non-autonomous UPR^MT^ signaling^29^. We used the *rgef-1p* neuronal promoter to drive FLP recombinase expression and inserted FRT sites in the introns of *ucr-2.3* at the genomic locus. While *ucr-2.3* containing FRT sites had slightly reduced cell non-autonomous UPR^MT^ signal, we found that *rgef-1p::FLP-*induced recombination of *ucr-2.3* did not further reduce the cell non-autonomous UPR^MT^ and thereby did not phenocopy the *ucr-2.3* mutation. This indicated that neuron-specific loss of *ucr-2.3* does not significantly impact cell non-autonomous UPR^MT^ signaling (Supp Fig 4Ai–ii). We were unable to use this system to test germline-specific knockout of *ucr-2.3* because germline-specific FLP/FRT recombination becomes ubiquitous in all tissues in the following generation^30^. Altogether, these data demonstrate that *ucr-2.3* primarily acts in the germline, not neurons, to mediate cell non-autonomous UPR^MT^ signaling.

We next asked how much of the *ucr-2.3* mitochondrial activity is confined to the germline. We tested this by using a temperature sensitive *glp-4(bn2)* mutation, which arrests germ cell proliferation at the restrictive temperature 25°C to create germline deficient adult animals^31^. First, at the restrictive temperature, 25°C, the *ucr-2.3* mutation still decreases mitochondrial respiration compared to wild-type animals. In addition, we find that germline deficient animals have decreased mitochondrial respiration compared to wild-type animals (Fig 4C), likely due to the loss of the high population of mitochondria in the germline^32^. However, mitochondrial respiration in germline deficient animals was not further reduced by the *ucr-2.3* mutation, indicating that the contribution of *ucr-2.3* to respiration is restricted to the germline (Fig 4C). In support of this, we found that mitochondrial respiration in germline deficient animals could be further reduced by RNAi knockdown of the ubiquitously expressed ETC subunit *cco-1* (Fig 4C).

We further find that *ucr-2.3* is essential for germline mitochondrial health and function. One of our initial observations of the *ucr-2.3* mutant was the reduced brood size and loss of proper germline development (Fig 4D, Supp Fig 4B–C). We found that germline rescue of *ucr-2.3* expression could completely restore the brood size deficiency we observed with *ucr-2.3* mutants, whereas neuronal rescue of *ucr-2.3* had no effect (Fig 4D). We also found that in *ucr-2.3* mutant animals, the UPR^MT^ transcript *hsp-6* is up-regulated in the gonad of *ucr-2.3* mutant animals (Fig 4E), suggesting that loss of *ucr-2.3* has created mitochondrial stress within the germline. Additionally, using a germline-specific mitochondrial morphology reporter strain^33^, we discovered that loss of *ucr-2.3* greatly impacts the morphology and content of germline mitochondria (Fig 4F). While wild-type animals have a dense network of mitochondria throughout the germline, the *ucr-2.3* mutation results in a more punctate and sparser mitochondrial network in the distal germ cells and especially oocytes (Fig 4Fi). In addition, the *ucr-2.3* mutation reduced average mitochondrial number or density of the mitochondrial network (Fig 4Fii) as well as an overall decrease in transcriptional fold change of mitochondrial genes compared to wild-type as observed by RNA-seq (Supp Fig 4E, Supp Table 1).

Interestingly, we did find that the average mitochondrial length increased with the *ucr-2.3* mutation (Fig 4Eiii). Considering that *ucr-2.3* mutants have compromised mitochondrial respiration presumably due to loss of complex III activity (Fig 2C, Fig 4D), this is in alignment with previous reports that defective mitochondria may fuse together as compensation to increase overall oxidative capacity^34^. In support of this model, we find that *ucr-2.3* mutant animals have higher uncoupled mitochondrial respiration compared to wild-type (Supp Fig 4D), despite their lower basal mitochondrial respiration (Fig 2C). These data indicate that *ucr-2.3* mutants have a higher spare respiratory capacity, a marker of mitochondrial stress adaptation^35^. Altogether, these data support a model in which *ucr-2.3* deficient mitochondria are stressed and fuse together to compensate for their reduced ETC function as a form of stress adaptation. We conclude that loss of *ucr-2.3* compromises the quality of germline mitochondria, which may lead to suppressed cell non-autonomous UPR^MT^.

We next asked whether cell non-autonomous UPR^MT^ signaling required a specific role of *ucr-2.3* or general germline mitochondrial integrity. We first tested whether we could recapitulate the UPR^MT^ suppression we observed with *ucr-2.3* mutants with the knockdown of other mitochondrial genes that shared comparable tissue-specific paralogs as *ucr-2.3*. Through searching the same transcriptomic databases, we found that the complex I subunit gene *gas-1* that is ubiquitously expressed also had a related paralog *nduf-2.2* that has enriched neuron/germline-expression analogous to the expression differences between *ucr-2.1/ucr-2.2* and *ucr-2.3* (Supp Fig 4F)^22–24^. When we generated an early stop mutation in the *nduf-2.2* genomic locus through CRISPR/Cas9 editing, we observed that similar to *ucr-2.3,* loss of *nduf-2.2* also suppressed cell non-autonomous UPR^MT^ signaling but had no effect on autonomous UPR^MT^ signaling (Fig 4G, Supp Fig 4G). This suggested that germline mitochondrial integrity may have a general role in cell non-autonomous UPR^MT^ signaling.

Second, we tested whether *ucr-2.3* mutant suppression of non-autonomous UPR^MT^ could be rescued by the germline-specific expression of the other *ucr-2* family genes, such as *ucr-2.1*. Using the same single copy mosSCI system, we integrated a wild-type copy of *ucr-2.1* driven by the *pie-1* germline-specific promoter into a mutant strain of *ucr-2.3* and observed that germline-specific expression of *ucr-2.1* could rescue non-autonomous UPR^MT^ in the *ucr-2.3* mutant (Fig 4H). Given the high sequence similarity between *ucr-2.1* and *ucr-2.3* and their proposed similar function in complex III of the ETC, this suggests that general mitochondrial quality in the germline is essential for non-autonomous UPR^MT^ signaling and that the tissue specific expression of *ucr-2.3* in the germline determines its special role in inter-tissue signaling.

Finally, we asked whether a more targeted germline mitochondrial dysfunction, beyond knockdown of mitochondrial proteins, could also affect cell non-autonomous UPR^MT^. We tested this by expressing a germline-specific mitochondrially-targeted KillerRed (mtKillerRed) (Supp Fig 4H), which generates reactive oxygen species upon light activation^36^. By activating mtKillerRed in early adulthood (Day 1), we could induce germline mitochondrial dysfunction after the full development and expansion of the germline in the *C. elegans* larval stages, and thereby decouple germline mitochondrial dysfunction from germline integrity (Supp Fig 4Hi). We observed that early adulthood activation of germline mtKillerRed suppressed cell non-autonomous UPR^MT^, indicating that germline mitochondrial quality itself is crucial for intact cell non-autonomous UPR^MT^ signaling (Supp Fig 4Hii–iii). We also observed a mild (but not statistically significant) suppression in cell non-autonomous UPR^MT^ without light activation of the mtKillerRed, perhaps due to low basal activity of KillerRed^37^. Altogether, these data indicate that general germline mitochondrial dysfunction inhibits neuron-derived cell non-autonomous UPR^MT^ signaling without altering autonomous UPR^MT^ induction.

### The germline is required for cell non-autonomous UPR^MT^ signaling

**Figure 5:**
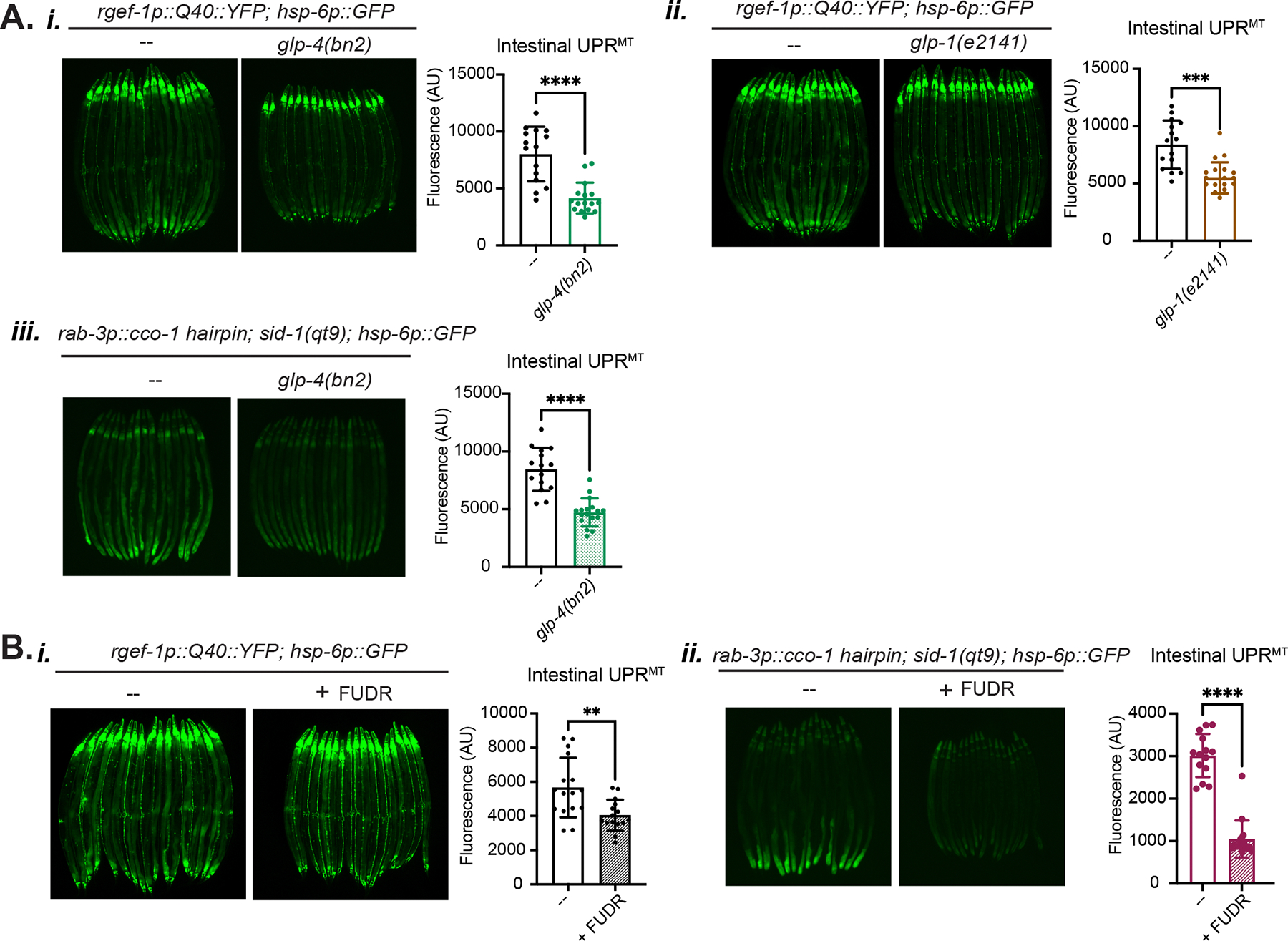
The germline is required for UPR^MT^ non-autonomous signaling. a. Comparison of fluorescence imaging and quantification of intestinal UPR^MT^ signal in germline deficient mutants at the restrictive temperature 25°C. ****, ***p<0.0001; n = 3. b. Comparison of fluorescence imaging and quantification of intestinal UPR^MT^ signal upon FUDR treatment. **p = 0.0034, ****p<0.0001; n = 3. See also Figure S5.

We next asked if the germline tissue itself was also essential for mediating cell non-autonomous UPR^MT^ signaling. To test this, we used genetic and pharmacological methods to deplete the germline (Fig 5). For a genetic method, we used the temperature-sensitive germline deficient mutations *glp-4(bn2)* and *glp-1(e2141ts)*, which restrict germ cell proliferation at the restrictive temperature of 25°C^31,38^, to ask whether germline deficient animals also have suppressed cell non-autonomous UPR^MT^ signaling (Fig 5A). Indeed, we observed that germline deficient animals suppress cell non-autonomous UPR^MT^ signaling in the neuronal PolyQ40 stress model as well as in the neuron-specific *cco-1* knockdown model (Fig 5A). Interestingly, the *glp-1(e2141ts)* allele is long-lived while *glp-4(bn2)* is not^39,40^, decoupling germline-mediated suppression of UPR^MT^ signaling from longevity mechanisms. Pharmacologically, we used the drug FUDR to ablate the germline through prohibiting DNA replication^41^ (Fig 5B, Supp Fig 5A). We found that FUDR treatment similarly suppressed cell non-autonomous activation of the UPR^MT^ in both the neuronal PolyQ40 and *cco-1* RNAi hairpin models (Fig 5B), further demonstrating that an intact germline is essential for cell non-autonomous UPR^MT^ signaling. In addition, we found that the genetic germline depletion via the *glp-4(bn2)* temperature sensitive mutation did not suppress the other cell non-autonomous signaling models for the UPR^ER^ or HSR (Supp Fig 5B). Overall, these results indicate that depletion of the germline tissue or germline mitochondria specifically inhibits cell non-autonomous UPR^MT^ stress signaling between neurons and the intestine.

### *ucr-2.3* mediates cell non-autonomous UPR^MT^ downstream of established neuronal factors in UPR^MT^ signaling

**Figure 6:**
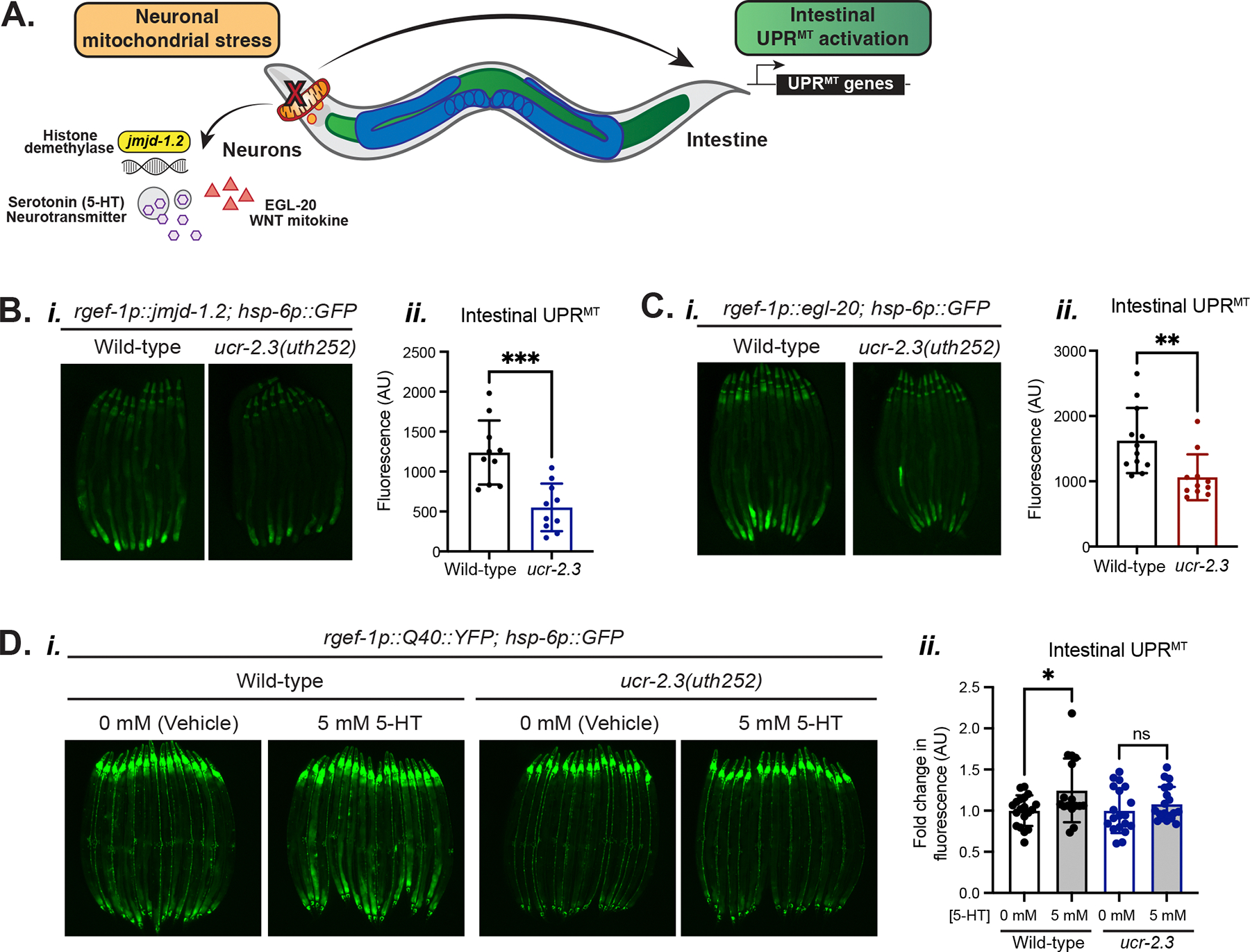
*ucr-2.3* operates downstream of established neuronal factors in mediating cell non-autonomous UPR^MT^ a. Known neuronal molecular players that mediate cell non-autonomous UPR^MT^ signaling in *C. elegans*. b. Fluorescence imaging comparison *(i)* and quantification *(ii)* of intestinal UPR^MT^ signal. ***p=0.0004; n = 3. c. Fluorescence imaging comparison *(i)* and quantification *(ii)* of intestinal UPR^MT^ signal. **p=0.0052; n = 3. d. Fluorescence imaging comparison *(i)* and quantification of fold change increase in intestinal UPR^MT^ signal *(ii)* for exogenous addition of serotonin (5-HT). *p = 0.0296, non-significant p-value = 0.3129; n = 4. See also Figure S6.

Our work demonstrated the critical role of germline mitochondria and germline-acting *ucr-2.3* in mediating UPR^MT^ signaling between neurons and the intestine. We next asked whether germline *ucr-2.3* functioned downstream of previously identified neuronal mitokine signaling factors^12,13,16^. Prior work had shown that neuronal overexpression of the Wnt ligand *egl-20* as well as the histone demethylase *jmjd-1.2* can activate intestinal UPR^MT^ even in the absence of neuronal mitochondrial stressors, such as neuronal PolyQ40 (Fig 6A)^13,16^. Thus, we tested whether the intestinal UPR^MT^ activation by neuronal over-expression of *egl-20* and *jmjd-1.2* required *ucr-2.3*. We found that loss of *ucr-2.3* suppressed intestinal UPR^MT^ activation by neuronal over-expression of *egl-20* and *jmjd-1.2*, suggesting that *ucr-2.3* in the germline functions downstream of these neuronal factors to regulate intestinal UPR^MT^ activation (Fig 6B–C).

Another neuronal mitokine factor we considered was serotonin, a neurotransmitter that is released by dense core vesicles and mediates cell non-autonomous activation of intestinal UPR^MT^ downstream of neuronal mitochondrial stress, such as PolyQ40^12^. We asked whether *ucr-2.3* acted up- or downstream of serotonin signaling by testing whether exogenous serotonin addition could rescue the suppressed cell non-autonomous UPR^MT^ in the *ucr-2.3* mutants. We found that while exogenous addition of serotonin (5-HT) enhances intestinal UPR^MT^ signaling in the PolyQ40 stress model as expected, loss of *ucr-2.3* prevented serotonin-mediated activation of intestinal UPR^MT^ (Fig 6D). Therefore, germline *ucr-2.3* functions downstream of neuronal factors *egl-20*, *jmjd-1.2*, and serotonin signaling to mediate intestinal UPR^MT^ activation upon neuronal mitochondrial stress.

Given that *ucr-2.3* functions downstream of these neuronal mitokine players, we considered the possibility that the germline directly receives mitokine signals of neuronal stress, such as serotonin and the Wnt ligand EGL-20, as a mechanism for mediating intestinal UPR^MT^ signaling (Supp Fig 6A). In this model (“Direct Signaling”), the germline would directly receive signals such as serotonin or Wnt/EGL-20, process them, and send a secondary signal to the intestinal to activate UPR^MT^. To test this model, we used a germline-specific RNAi strain to knockdown Wnt and serotonin receptors and asked if any germline-specific activity of these receptors mediated UPR^MT^ signaling. However, we found that germline-specific RNAi knockdown of these receptors had little impact on UPR^MT^ signaling (Supp Fig 6B–C), suggesting that the germline tissue itself does not directly receive these neuronal factors to mediate cell non-autonomous UPR^MT^. Additionally, we found that germline RNAi-specific knockdown of UPR^MT^ regulators such as the transcription factor *atfs-1*, the histone demethylase *jmjd-1.2*, and the Wnt mitokine itself *egl-20* does not impact cell non-autonomous UPR^MT^ signaling (Supp Fig 6D). Altogether, these data suggest that *ucr-2.3* in the germline has an independent and downstream role from these neuronal mitokine factors in mediating cell non-autonomous UPR^MT^ activation in the intestine.

### Germline mitochondria regulate intestinal UPR^MT^ activation by altering lipid metabolic and transport pathways

Our data indicate that *ucr-2.3* in the germline works downstream of the neuronal factors in regulating intestinal UPR^MT^ activation but does not directly receive neuronal factors. Therefore, we considered a different model of signaling in which the germline works in parallel with the neuronal factors to coordinate UPR^MT^ induction in peripheral cell types, such as the intestine. In this model (“Parallel Signaling”), a signal is sent from the germline to work with neuronal-derived Wnt signaling factors in peripheral cells to specifically induce the UPR^MT^ and not developmental programs mediated by canonical Wnt signaling.

**Figure 7:**
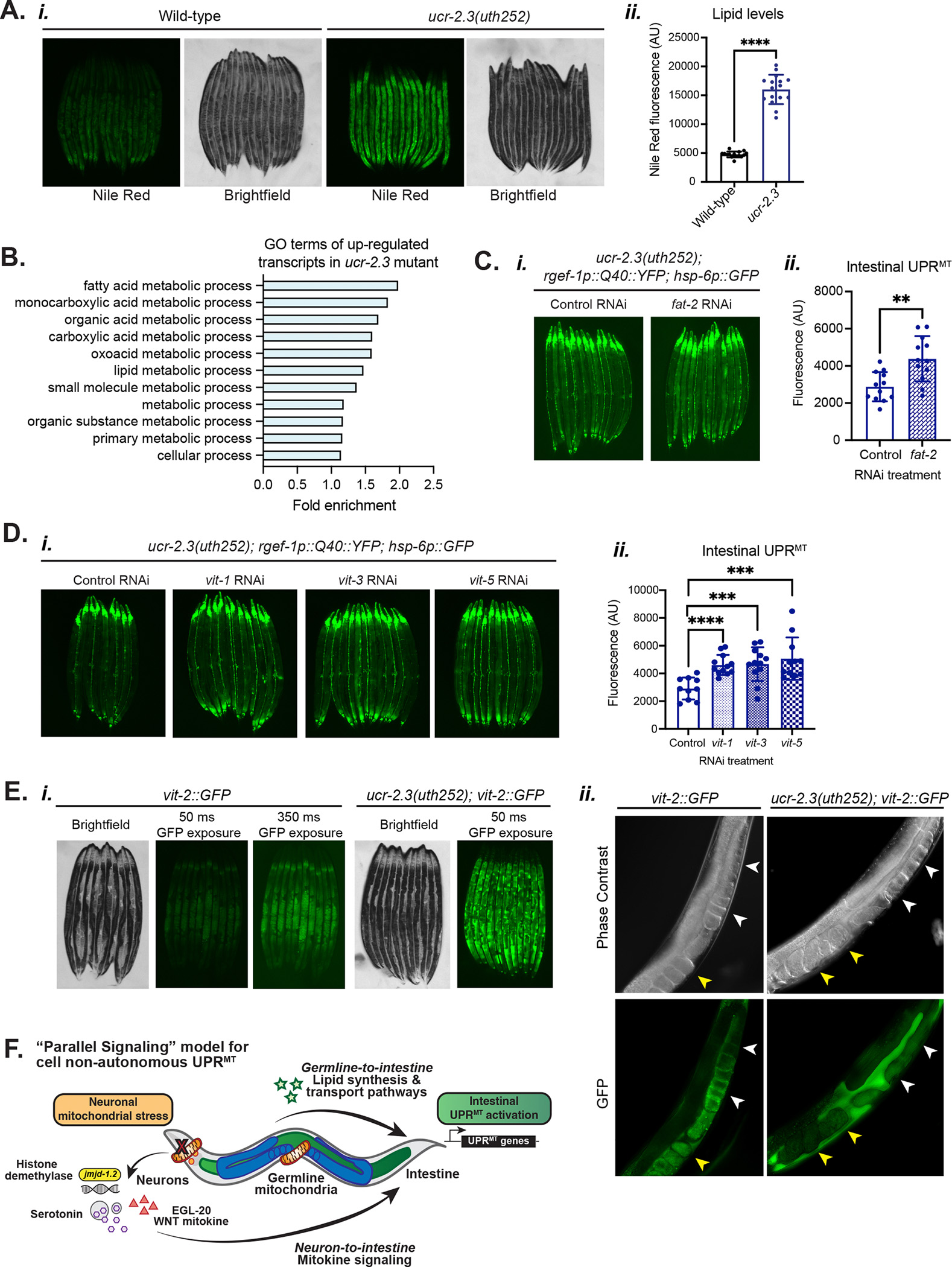
The germline regulates intestinal UPR^MT^ activation in the intestine through alteration of lipid synthesis and transport pathways. a. Nile red staining *(i)* and quantification *(ii)* of intestinal lipid levels. ****p <0.0001; n = 3. b. Enriched GO terms (biological process) in up-regulated transcripts in the *ucr-2.3(uth252); rgef-1p::Q40::YFP* mutant compared to *rgef-1p::Q40::YFP* alone. Shown are Fold enrichment values determined by the PANTHER statistical overrepresentation test. c. Fluorescence comparison *(i)* and quantification *(ii)* of intestinal UPR^MT^ signal. **p = 0.0020; n = 2. d. Fluorescence comparison *(i)* and quantification *(ii)* of intestinal UPR^MT^ signal. ****p < 0.0001, ***p < 0.001; n > 3. e. Comparison of vitellogenin lipoprotein levels and tissue localization. *(i)* Low magnification imaging at different levels of GFP exposure to illustrate differences in vitellogenin lipoprotein levels. *(ii)* High magnification comparison of vitellogenin localization. White and yellow arrowheads indicate location of oocyte and embryos, respectively. f. Parallel Signaling model for how germline mitochondria mediate neuron-to-intestine UPR^MT^ stress signaling. See also Figure S7.

To identify which signals could be sent from the germline to regulate UPR^MT^ induction in the intestine, we considered several established signaling pathways between the germline and the somatic tissues^42–47^. We were motivated to investigate a lipid signaling model between the germline and intestine for several reasons. First, we had observed that the *ucr-2.3* mutant had significantly higher levels of intestinal fat compared to wild-type (an approximately 3-fold increase as measured by Nile Red fluorescence) (Fig 7A, Supp Fig 7A–B). In addition, many of the up-regulated transcripts in the *ucr-2.3(uth252)* mutant animals by RNA-seq were highly enriched in gene ontology (GO) terms for metabolic processes involving fatty acids and lipids (Fig 7B, Supp Fig 7E). We confirmed that this increase in intestinal fat was not due to increased food consumption, as *ucr-2.3* mutant animals had slightly reduced feeding rates compared to wild-type animals (Supp Fig 7D).

We postulated that the increased intestinal lipid content of *ucr-2.3* mutants may be due to its germline-specific role. To test this, we measured how loss of the other *ucr-2* family genes *ucr-2.1* and *ucr-2.2*, which do not have germline-restricted expression and do not regulate intestinal UPR^MT^, affect intestinal lipid content. We found that loss of either *ucr-2.1* or *ucr-2.2* had minimal impact on intestinal fat levels, and the double knockdown of *ucr-2.1/ucr-2.2* decreased intestinal fat levels (Supp Fig 7C). Separately, we found that germline deficient mutant animals, which have suppressed intestinal UPR^MT^ (Fig 5A) similar to *ucr-2.3* mutants, also have higher intestinal fat levels (Supp Fig 7F), similar to previous observations^48^. Overall, these data suggested that increased intestinal lipid levels of *ucr-2.3* mutant animals derive from their compromised germline integrity and could lead to the suppression of intestinal UPR^MT^ activation.

Thus, we next asked whether we could rescue the suppressed intestinal UPR^MT^ activation in *ucr-2.3* mutants through altering intestinal lipid metabolic pathways. We conducted a targeted RNAi screen of fatty acid and lipid metabolic genes up-regulated in the *ucr-2.3(uth252)* mutant (Fig 7B) and asked if knockdown of these lipid metabolic pathways could rescue suppressed UPR^MT^ in the *ucr-2.3* mutant. We found that knockdown of *fat-2*, a Δ12 desaturase enzyme that converts oleic acid to linoleic acid in the first step of polyunsaturated fatty acid (PUFA) synthesis in *C. elegans*^49,50^, rescued non-autonomous UPR^MT^ signaling in the *ucr-2.3(uth252)* mutant (Fig 7C). This suggested that the increased fatty acid synthesis and lipid levels in the intestine of germline-deficient, *ucr-2.3* mutant animals lead to suppressed intestinal UPR^MT^.

We next asked how germline-deficient, *ucr-2.3* mutant animals could lead to increased intestinal fat levels by investigating a lipid transport pathway between the intestine and the germline mediated by the *vit* family of vitellogenin lipoproteins^51^. The *vit* family vitellogenins transport phospholipids, cholesterol, and other nutrients between the intestine and the germline by binding to lipids in the intestine, secrete as yolk lipoproteins into the pseudocoelom, and are taken up by oocytes in the germline through receptor mediated endocytosis^52^. We found that the *vit* family genes (*vit-1, 2, 3, 5, 6*) are all transcriptionally up-regulated in our *ucr-2.3* mutant RNA-seq dataset (mean log_2_(FC) = 0.669, SD = 0.191), similar to what we observed for lipid synthesis genes (Supp Fig 7E). Additionally, we found that RNAi knockdown of the *vit* family genes, which redistributes lipids and increases lysosomal lipolysis in the intestine^53^, rescued intestinal UPR^MT^ activation in the *ucr-2.3* mutant (Fig 7D). Collectively, our data suggest that the synthesis or transfer of lipids between the intestine and germline operates downstream of germline integrity to regulate UPR^MT^ activation in the intestine.

We next asked how intestine-to-germline lipid transport was altered in the *ucr-2.3* mutant animals. Using the *vit-2::GFP* reporter strain to monitor secretion of vitellogenins from the intestine to the germline, we observed two striking phenotypes with the *ucr-2.3* mutation (Fig 7E). First, compared to wild-type animals, the *ucr-2.3* mutant animals had dramatically up-regulated levels of VIT-2::GFP (Fig 7Ei), consistent with our RNA-seq data and overall higher intestinal lipid levels in the *ucr-2.3* mutant (Fig 7A, Supp Fig 7A–B, E). Second, while wild-type animals had properly localized VIT-2::GFP lipoprotein to oocytes (white arrowheads) and embryos (yellow arrowheads) in the germline, *ucr-2.3* mutant animals had perturbed VIT-2 lipoprotein localization (Fig 7Eii). For the *ucr-2.3* mutant animals, the VIT-2::GFP was mostly observed in the pseudocoelom of the animals and excluded from the germline, suggesting that in *ucr-2.3* mutant animals, the VIT-2 lipoprotein can be secreted from the intestine but is not properly endocytosed into the oocytes and the resulting embryos. We hypothesized that this may result from fewer oocytes and less developed female germline of the *ucr-2.3* mutant animals since the proximal oocytes are the primary receiving cells for vitellogenin transport from the intestine^51^. Indeed, as we had found that hermaphrodite *ucr-2.3* animals had a large increase in intestinal lipid levels (3.38x fold increase compared to wild-type hermaphrodites), we also found that *ucr-2.3* mutant males that lack the female germline have a much smaller increase in intestinal lipids (1.28x fold increase compared to wild-type males) (Supp Fig 7G). These data suggest that the majority of the increased intestinal lipid content in *ucr-2.3* mutant animals derives from the lack of proper oocyte proliferation and female germline development (Supp Fig 4C). Altogether, these data suggest that the lack of proper oocyte and germline development in *ucr-2.3* mutant animals perturbs intestine-to-germline vitellogenin-based lipid transport and leads to an over-accumulation of lipids in the intestine, which suppresses UPR^MT^ activation.

## Discussion

In this study, we have identified the importance of the germline, and particularly of germline mitochondria, in mediating cell non-autonomous communication of mitochondrial stress from the nervous system to the periphery. In this model (“Parallel signaling”), cell non-autonomous activation of the intestinal UPR^MT^ integrates signals in parallel from the nervous system (e.g. Wnt and serotonin) *and* from an intact, healthy germline, perhaps through a particular lipid species that becomes dysregulated upon germline depletion (Fig 7F). Our discovery helps to resolve a previous conundrum presented by the control of cell non-autonomous UPR^MT^ induction by Wnt ligand signaling without the corresponding Wnt role in development^16^.

An important aspect of our model is the lipid-based signaling between the germline and the intestine for UPR^MT^ activation. This is based on our findings that *ucr-2.3* mutant animals that suppress intestinal UPR^MT^ have higher intestinal lipid levels and dysregulated intestine-to-germline lipid transport (Fig 7A, B, E; Supp Fig 7E). This dysregulated lipid transport is likely due to the compromised germ cell mitochondrial function in the *ucr-2.3* mutant, which has fewer developed oocytes to receive vitellogenin-bound lipids from the intestine (Fig 4D, Supp Fig 4B–C, Fig 7E). The lack of developed oocytes results in an aberrant accumulation of intestinal lipids, compromised intestine-to-germline lipid transport, and suppression of cell non-autonomous UPR^MT^ in the intestine. Given the strong up-regulation of the VIT-2 lipoprotein in the *ucr-2.3* mutant (Fig 7E), it is also possible that the *ucr-2.3* mutant germline can sense compromised lipid import and send a compensatory signal to the intestine to further up-regulate lipid synthesis and vitellogenin lipid transport in a positive feedback loop. Whether this compensatory signal is mediated by the compromised mitochondria in *ucr-2.3* mutant oocytes is an intriguing possibility and warrants further exploration. Additionally, while our data suggest that the accumulation of neutral intestinal lipids leads to suppression of UPR^MT^ (Fig 7C–D), there is likely a more specific, and so far unidentified, lipid species that regulates intestinal UPR^MT^ activation.

Beyond the “Parallel Signaling” model, we also considered a “Direct Signaling” model, where the germline could receive mitokine signals secreted by stressed neurons, process these signals – perhaps through mitochondrial *ucr-2.3* activity – and emit a secondary signal to the intestine to regulate UPR^MT^ (Supp Fig 6A). While we were unable to identify a germline-specific receptor involved in receiving neuronal mitokine signals in support of a Direct Signaling model (Supp Fig 6B–C), work from others suggests possible mechanisms for direct neuron-to-germline stress communication. For instance, in the same neuronal PolyQ40 model of mitochondrial stress in *C. elegans*, neuronal mitochondrial stress elevated mtDNA levels in germline mitochondria, which could be transgenerationally inherited^54^. Furthermore, components of the Wnt signaling pathway, such as the β-catenin *bar-1,* were also shown to directly regulate oocyte mitochondria mass and mtDNA levels^54^. Serotonin signaling has also been shown to directly improve oocyte quality in *C. elegans* and *D. melanogaster*^55^. Whether a Direct Signaling route between neurons and germ cells could exist in the context of PolyQ40 or other forms of neuronal stress should be explored in future studies.

Our data suggest that *ucr-2.3* directs mitochondrial signaling through its canonical ETC role in mitochondrial activity and that the unique role of *ucr-2.3* in inter-tissue signaling derives from its germline-specific expression. Additionally, since other germline restricted ETC genes, such as *nduf-2.2*, were also required for inter-tissue UPR^MT^ signaling (Fig 4G), we speculate that evolution has opted to restrict expression of a few, key ETC genes within the germline to help coordinate signals to the soma. Whether mitochondrial ETC isoforms with tissue-specific expression also exist in humans should be further explored. It is also worthwhile to consider whether there may also be an additional, non-ETC role for *ucr-2.3* in generating a signal for germline-to-intestine UPR^MT^ signaling. The UCR-2.3 protein sequence is predicted to belong to the M16 metallopeptidase family, which includes mitochondrial processing peptidases (MPP). Indeed, the mammalian ortholog of *ucr-2.3*, UQCRC2, is predicted to have MPP-like protease activity in combination with UQCRC1 to cleave the N-terminal MTS of UQCRFS1 (the Rieske Fe-S protein) in the final steps of complex III assembly^56–58^. Whether *C. elegans* UCR-2.3 could also have an MPP-like or a general peptidase role in generating peptide-like signals in the context of mitochondrial stress is an interesting possibility.

Our findings highlight an important role of the germline and germline mitochondria in mediating stress signaling pathways with somatic tissues. We propose that this specialized role for germline mitochondria may derive from the importance of mitochondrial quality for germline maintenance. Because of the high abundance of mitochondria in germ cells such as oocytes and the importance of mitochondrial quality in germ cell maturation^59,60^, the integrity of the germline and its communication with other tissues in the soma, may have a heavy reliance on mitochondrial function. More broadly, we suggest there may be an evolutionary basis for this germline signaling circuit to conserve organismal resources in the somatic tissues. The need to activate energy-intensive, protective stress responses like the UPR^MT^ should only be executed in the presence of a functioning germline to preserve progeny production and continuation of the immortal germline. Without a functional germline, there would be no need to activate these energy-intensive stress responses, and the UPR^MT^ is shut off to direct resources elsewhere. A further understanding of this signaling circuit will inform how germ cell and germline mitochondrial quality may be influenced by stresses occurring in the somatic environment, such as with aging or metabolic disease. This may be critical for understanding what consequences stresses occurring in the somatic tissues may have on germline mitochondrial quality, fertility or reproductive tissue health, and lipid metabolism, especially given existing connections between obesity and sterility/infertility in humans^61–63^.

### Limitations of the Study:

It is difficult to over-express or knock genes down specifically in the germline without the genetic perturbation becoming ubiquitous in following generations. Our ability to knockdown mitochondrial function in the germline was mostly enabled by our discovery of the germline-specific role of *ucr-2.3*. Also, given the tight linkage between germline mitochondrial function and germline quality, we cannot completely isolate germline-specific mitochondrial dysfunction from germline integrity. Finally, our study only investigates the role of germline signaling in *C. elegans*. A fuller context of the role of germline and oocyte mitochondrial quality control in stress signaling for human health it will be important will require extending this work into mammalian species.

## STAR Methods

### RESOURCE AVAILABILITY

#### Lead contact

Further information and requests for resources and reagents should be directed to and will be fulfilled by the lead contact, Dr. Andrew Dillin (dillin@berkeley.edu).

#### Materials Availability

All *C. elegans* strains and cell lines used in this study are available by direct request to the lead contact, Dr. Andrew Dillin (dillin@berkeley.edu).

#### Data and Code Availability

- RNA-seq datasets are available in Table S1 and deposited in Mendeley (doi:10.17632/hpbddm6k5p.2). All data reported in this paper will be shared by the lead contact, Dr. Andrew Dillin (dillin@berkeley.edu), upon request.
- This manuscript does not report original code.
- Any additional information required to reanalyze the data reported in this paper is available from the lead contact upon request.

### EXPERIMENTAL MODEL AND STUDY PARTICIPANT DETAILS

#### *C. elegans* strain generation and maintenance

*C. elegans* animals were fed standard OP50 bacteria and grown on standard NGM containing media. For all RNAi knockdown experiments, *C. elegans* animals were fed HT115 bacteria expressing dsRNA targeted to the gene of interest on NGM media containing IPTG and appropriate antibiotics for selection of the dsRNA-containing plasmid. All strains were either generated by this study, generated by SUNY Biotech, or ordered from the Caenorhabditis Genetics Center (CGC). The only exceptions are the SJZ106 strain (*foxSi27[pie-1p::tomm20::mKate2::HA::tbb-2 3’UTR]*), which was a kind gift from Dr. Steve Zuryn (The University of Queensland), and the APW202 strain (*ucr-2.3::GFP*), which was a kind gift from Dr. Andrew Wojtovich (University of Rochester). CRISPR-edited strains were either ordered from SUNY Biotech or generated by this study following standard *C. elegans* CRISPR/Cas9 genome editing protocols^65^ and the Alt-R CRISPR-Cas9 system (IDT). Briefly, Cas9 protein (UC Berkeley QB3 MacroLab) was combined with Alt-R CRISPR tracrRNA, a crRNA mixture (targeted to our gene of interest and *dpy-10*), and ssDNA repair template mixture (again, targeted to our gene of interest and *dpy-10*) as a ribonucleoprotein complex and injected into the gonad of L4 stage *C. elegans* hermaphrodites. This allowed us to select “jackpot” broods containing many F1 animals with “roller” or “dumpy” phenotypes to improve efficiency in selection of broods with edited genomes. mosSCI integration of strains were generated following standard procedures^28^. Briefly, constructs of interest were cloned into the pCFJ356 mosSCI plasmid backbone. A DNA mixture of the mosSCI plasmid of interest, the pCFJ601 plasmid (containing the mosSCI transposase), and mCherry expressing plasmids (to eliminate non-integrated, array animals) was injected into the gonad of young, “uncoordinated” EG6703 animals. Resulting progeny were screened for broods that yielded only “mover” animals with no mCherry-expressing arrays.

#### Cell culture lines, virus generation and transduction

Human hTERT-RPE-1 PAC knockout cells (gifted by Andrew Holland ^66^) were grown in DMEM:F-12 (Gibco) supplemented with 10% FBS (VWR Scientific), 1% Glutamax (Gibco), 1% NEAA (Gibco), and 1% Penicillin/Streptomycin (Gibco). HEK-293T cells were grown in DMEM (GIBCO) containing 10% FBS (VWR Scientific), 1% Glutamax (Gibco), 1% NEAA (Gibco), and 1% Penicillin/Streptomycin (Gibco). Cells were maintained at 37° C with 5% CO_2_ at atmospheric O_2_. Cultures were passaged enzymatically every 3–4 days using 0.25% Trypsin (Gibco) diluted 1:3 in 1X PBS (Gibco). Trypsin was inactivated by cell media.

### METHOD DETAILS

#### Animal synchronization

Unless stated, all *C. elegans* experiments were performed with synchronization using a standard hypochlorite treatment protocol. Briefly, animals were washed off NGM plates with a M9 solution and bleached with 1.8% sodium hypochlorite solution until all animal carcasses were disintegrated. Resulting eggs were washed five times with M9 solution using centrifugation and eggs were plated on respective plates for the experiments. All experiments were conducted at 20 °C except for germline ablation experiments with temperature sensitive mutants, which were done at the restrictive temperature of 25 °C as indicated in figure legends.

#### Mutagenesis screen

*rgef-1p::Q40::YFP; hsp-6p::GFP* animals at the L4 stage were incubated with Ethyl-methyl sulfonate (EMS, Sigma). Five plates of 250 F1 progeny each were selected to screen 2500 genomes. F2 progeny were screened for suppression of *hsp-6p::GFP* signal in the intestine. Identified suppressors were singled onto fresh plates and further screened for homozygous, recessive mutations. Selected suppressor mutants were backcrossed to the parental strain carrying the reporter and polyQ transgenes at least one time.

#### Mutation mapping

We followed a SNP-based whole genome sequencing method to identify putative causative mutations in the suppressor mutants^17^. Briefly, we outcrossed suppressor mutant strains with males from a Hawaiian CB4866 strain containing the *rgef-1p::Q40::YFP* and *hsp-6p::GFP* transgenes. F2 progeny containing the suppression phenotype were selected and allowed to self-fertilize for two generations. Then, genomic DNA was extracted from these suppressor lines using the DNeasy kit (Qiagen) and sent to the Beijing Genomics Institute for WGS on their Illumina HiSeq platform using single 90 nucleotide reads. Genomic data was analyzed using MAQgene as previously described^17,67^.

#### Fluorescence stereoscope imaging and quantification

Animals were synchronized by hypochlorite treatment and hatched on either control or respective HT115 RNAi bacteria. Animals were grown to Day 2 at 20 °C unless otherwise indicated, like for the restrictive temperature experiments. For imaging experiments in the *rab-3p::hsf-1 OE; hsp-16.2p::GFP* background, animals were heatshocked for imaging as had been previously published ^68^. Briefly, animals were hatched and allowed to develop at either 20 °C or 25 °C (if a temperature sensitive mutant). On the day of the experiment, animals were then heatshocked at 34 °C for 2 hr, then let recover at their original temperature they developed at for 2 hr, then imaged. For imaging, Day 2 animals were anesthesized with 0.1 M sodium azide and lined up on an NGM plate without a bacterial lawn. Animals were imaged using M250FA stereoscope (Leica) under respective fluorescence. Animals were imaged at 250 ms exposure under GFP excitation fluorescence unless otherwise indicated. Quantification of intestinal GFP signal was performed using ImageJ/FIJI by tracing the intestinal regions. Statistical analyses were performed with GraphPad PRISM as described in the figure legends.

#### Quantitative RT-PCR

Synchronized animals were collected on Day 2 of adulthood and gravity washed in M9 buffer to isolate adults and remove larvae. Animals were collected in three independent biological replicates for one experiment. Washed animals were then frozen in TRIzol (Invitrogen) and freeze thawed using liquid nitrogen. RNA was harvested from animals using a TRIzol-based extraction method and RNA was purified using a RNeasy Mini Kit (Qiagen). cDNA was synthesized using the QuantiTect Reverse Transcription Kit (Qiagen) using equivalent amounts of RNA per sample. qPCR was performed using a standard curve protocol using SYBR Select Master Mix (Life Technologies). Statistical analyses were performed with GraphPad PRISM as described in the figure legends.

#### Sub-cellular fractionation and western blotting

Sub-cellular fractionation and mitochondrial enrichment of *C. elegans* lysates was performed as previously described. Briefly, synchronized animals were mechanically homogenized with a Dura-Grind Stainless Steel Dounce Trissue Grinder (Wheaton) in mitochondrial extraction buffer (5 mM Tris-HCl / 7.4, 210 mM mannitol, 70 mM sucrose, and 0.1 mM EDTA) with protease inhibitor. Lysates were subject to differential centrifugation. Protein concentration was measured using a Rapid Gold BCA kit (Pierce) before being loaded onto Tris-Glycine 4–12% gels (Invitrogen). Gels were transferred using NuPAGE transfer buffer (Invitrogen) onto nitrocellulose membranes (Biorad) and blocked with LI-COR Blocking Buffer (LI-COR) with Tween. Membranes were probed with antibodies targeted to HA (Abcam, ab9110), NDUFS3 (Abcam, ab14711), and a Tubulin (Sigma, T9026).

#### Cloning of plasmids for cell culture

*C. elegans* (human codon-optimized) UCR-2.3-mNeonGreen and OMP25-tagBFP2 were synthesized as geneblocks (IDT), PCR amplified with Q5 polymerase (New England Biolabs) and incubated with restriction enzyme-digested CD510-B1 plasmid (SystemBio) and assembled with Gibson Assembly Master Mix (New England Biolabs).

#### Generation and transduction of UCR-2.3 lentivirus

Lentiviral plasmids encoding pCMV-OMP25-tagBFP2-EF1a-NEO and pUbC-UCR-2.3-mNeonGreen-EF1a-Puro were transfected into HEK-293T cells with lentiviral packaging plasmids with Lipofectamine 3000 (Invitrogen) according to standard lentiviral production protocols. Viral supernatant was filtered through a 0.45 μm filter before transduction. RPE-1 cells were transduced with lentivirus at an MOI of 0.3–0.5 (usually between 50–100 μL of virus/well) and 10 μg/mL Polybrene (Sigma-Aldrich). After 48 hours, RPE-1 cell media was replaced and grown in the presence of 1 μg/mL Puromycin (Gibco) or 100 μg/mL Geneticin (Gibco) for about 7 days and until all non-transduced control cells died.

#### Live cell microscopy of human cells

RPE-1 cells were seeded at 1.25 × 10^5^ cells/dish onto Fibronectin-coated plates, 35-mm glass-bottom dishes with #1.5 cover glass (Cellvis) 24 hours prior to imaging. Prior to live cell imaging, cell medium was replaced with pre-warmed FluoroBrite DMEM (Gibco) supplemented with 10% FBS, 1% Glutamax, 1% NEAA, 1% Penicillin/Streptomycin, 1 μM SYTO Deep Red Fluorescent Nuclei Acid Stain (Invitrogen), and 1 μM Probenecid (Invitrogen). Imaging of live cells was performed with a Zeiss Airyscan microscope LSM900 equipped with heated stage and environmental chamber maintained at 37° C and 5% CO_2_. Images were acquired using the ZenBlue3.1 software followed by deconvolution and Airyscan processing, and further processed using ImageJ/FIJI. Mander’s colocalization coefficients are calculated using the Colocalization threshold plug-in in ImageJ, in which the thresholds were set by the Costes Auto threshold method.

#### Mitochondrial oxygen consumption assays

All mitochondrial oxygen consumption rate assays (OCR) were performed using a Seahorse XFe96 Analyzer (Agilent) at 20 °C following a previously published protocol. Briefly, the sensor cartridge was calibrated the day before at room temperature, and appropriate drugs (NaN_3_, FCCP) were loaded into the injection ports. Day 2 adult animals were gravity washed with M9 buffer, then loaded 10–20 animals per well into the cell culture plate. After respirometry run was completed, the number of animals were counted per well to normalize the data. Statistical analyses were performed with GraphPad PRISM as described in the figure legends.

#### Fluorescence widefield microscopy of *C. elegans*

Slides for imaging were prepared by making a fresh flattened 5% agarose pad. Animals were immobilized in 12 μl of 0.1% NaN_3,_ then sealed beneath a 22×22mm coverglass. 6–10 animals were imaged per condition per experiment. Imaging was done using a Leica DM6 Thunder Imager and processed using LAS X software automated Thunder small volume computational clearing.

#### Brood size assay

For each strain tested, five L4 animals were singled onto OP50 bacterial plates and kept at 20 °C to lay. Animals were moved each day to new OP50 plates until Day 5. Progeny laid by each singled animal were counted at the L4 or Day 1 life stage. Statistical analyses were performed with GraphPad PRISM as described in the figure legends.

#### Quantification of mitochondrial features using FIJI

To quantify mitochondrial number and size in images of mitochondrial reporter worms, Z-stack images were first projected into a single layer using the “Average Intensity” function. The “Auto Local Threshold” function was applied with parameters of “method=Bernsen radius=15 parameter_1=0 parameter_2=0 white” to define mitochondria. The “Skeletonize (2D/3D)” function was applied to extract individual mitochondrion and the “Analyze Skeleton (2D/3D)” function was applied to count the number of these mitochondrial skeletons. To calculate mitochondrial density, the area of the whole germline region was manually selected and measured using FIJI (1 pixel area=0.010645 μm^2^).

#### Gonad extraction for RT-PCR analysis

Approximately 6–10 Day 2 adult worms were picked into a 50μl droplet of Egg Buffer (25mM HEPES pH7.3, 118 mM NaCl, 48mM KCl, 2mMCaCl_2_, 2mMMgCl_2_) + 0.01% Tween20+ 0.01% tetramisole on a glass slide. Worms were decapitated below the pharynx using a feather scalpel, allowing the gonads to self-extrude. Gonads were separated from body using two syringes and collected in a sterile 1.5 ml microfuge tube and flash frozen in liquid nitrogen. RNA was extracted using Qiagen RNeasy Micro Kit (Cat.740004). cDNA synthesis was performed using Qiagen QuantiTect Reverse Transcription Kit (Cat. 205311).

#### KillerRed light activation

mtKillerRed strain was generated through mosSCI integration as described above. The mtKillerRed mosSCI plasmid was constructed in the pCFJ356 backbone with the KillerRed construct fused N-terminally to the first 50 amino acids of *tomm-20* (as a mitochondrial targeting sequence) and with the 3’UTR of *tbb-2*. For light activation, strip 0.2 μl PCR caps were filled with 30μl of 1% agarose and allowed to solidify. 25μl M9 was added to the caps and 10–12 Day 1 animals were added. KillerRed expressing and control worms were irradiated under 545 nm green light for 10 minutes. Worms were recovered on OP50 plates and imaged 24 hours later.

#### Exogenous serotonin addition

Exogenous serotonin addition was performed following a published procedure ^12^. Serotonin hydrochloride (Sigma) was solubilized in MilliQ water and plated onto NGM agar plates already containing control HT115 RNAi bacteria. Solubilized serotonin concentrations were calculated so that the final serotonin concentration as stated considers the total agar volume of the plate (approximately 9.9 ml). Seeded serotonin plates were allowed to dry overnight in the dark and plated with eggs harvested from a hypochlorite treatment for the experiment.

#### 5-Fluoro-2’deoxyuridine (FUDR) treatment

Eggs were harvested from hypochlorite treatment of animals and plated on control HT115 bacterial plates. 100 μL of 10 mg/mL FUDR was seeded onto the control HT115 bacterial lawn and let dry overnight at room temperature. At the L4 developmental stage, animals were moved onto the FUDR-treatment plates and let grown to Day 2 of adulthood for their respective experiment.

#### Oil Red O and Nile Red staining

Lipid staining using Oil Red O or Nile Red was conducted following previously published protocol^70^. Briefly, synchronized animals were grown to Day 2 on respective HT115 RNAi media. Animals were washed in PBS-T, fixed in 40% isopropanol, then incubated with Oil Red O or Nile Red stain for 2 hours at room temperature shielded from light. Excess dye was washed off and animals were imaged via fluorescence stereoscope imaging for Nile Red using a M250FA stereoscope (Leica) or brightfield/colorimetric imaging for Oil Red O using a Revolve microscope (Echo). Quantification of signal was performed in ImageJ by tracing intestinal regions for fluorescent Nile Red images or Oil Red O colorimetric images. For quantification, Oil Red O images were transformed into the HIS color space using the Color Transformer 2 plugin and quantified in the (S) channel. Statistical analyses were performed with GraphPad PRISM as described in the figure legends.

#### Pumping assay

To assess pumping rate, synchronized animals were grown to Day 2 on respective HT115 RNAi media. 10 animals per condition were plated onto individual plates 2 hrs before the assay to allow acclimation to the plate and avoid alterations to their movement and feeding rates. Pumps per animal were counted for 30 secs each and repeated 3 times per animal as a technical replicate, and pump rate was calculated as an average across the 10 animals. Statistical analyses were performed with GraphPad PRISM as described in the figure legends.

#### RNA-seq library preparation and analysis

RNA was prepared using methods similar to those for RT-qPCR as described above. RNASeq library preparation was performed using Roche products, KAPA Biosystems mRNA HyperPrep Kit and KAPA Unique Dual Index Adapters. Thermo Fisher Scientific NanoDrop and Qubit instruments were used to measure nucleic acid concentrations. Agilent BioAnalyzer was used to determine initial Total RNA and RNASeq library Quality. Single direction sequencing was performed using an Illumina NovaSeq, mode SP, SR100 at the Vincent J. Coates Genomic Sequencing Core at University of California, Berkeley. RNASeq analysis was performed by uploading .fastq files to the Galaxy web platform^71^, using the public server at usegalaxy.org and the *C. elegans* FASTA reference transcriptome Caenorhabditis_elegans.WBcel235.cdna.all.fa, downloaded from ensembl. Tools included Kallisto Quant v0.48.0+galaxy1 and DESeq2, v2.11.40.8+galaxy0^72^. GO term enrichment analysis was conducted using the PANTHER Overrepresentation Test on geneontology.org using genes significantly up-regulated with log_2_(FC) > 1. Only results with FDR p value < 0.05 were considered.

### QUANTIFICATION AND STATISTICAL ANALYSIS

The Prism 10 software (GraphPad) was used for statistical analyses. Data are represented as mean ± SD. Unpaired two-tailed t Test assuming Gaussian distribution with Welch’s correlation (not assuming equal variance) was used for all comparisons unless otherwise noted. Independent biological replicate experiments are noted as “n=” for all data shown. P-values were used to quantify the statistical significance of the tests.

## Supplementary Material

Supplementary Fig 1 Supplementary Figure 1: Additional genetic evidence supporting loss of function mutations in *ucr-2.3* suppress cell non-autonomous UPR^MT^ signaling in *C. elegans*, Related to Figure 1
a. Fluorescence comparison of intestinal UPR^MT^ signal in the *rgef-1p::Q40::YFP;hsp-6p::GFP* genetic background with and without the EMS mutant allele obtained in the suppressor screen *uth16*. n > 3.
b. Fluorescence comparison of intestinal UPR^MT^ signal with and without a loss of function mutation *ucr-2.3(uth214)* generated by CRISPR/Cas9 genome editing. n > 3.
c. Fluorescence imaging *(i)* and quantification *(ii)* of intestinal UPR^MT^ signal with and without a large deletion mutation *ucr-2.3(ok3073)*. p = 0.0010; n > 3.
d. qRT-PCR comparison of *ucr-2.3* expression levels in wild-type and *ucr-2.3(uth252)* mutant animals in the *rgef-1p::Q40::YFP; hsp-6p::GFP* genetic background. Transcript levels normalized by *rpl-32*. **p=0.0016; n = 3.
e. Fluorescence imaging showing autonomous UPR^MT^ activation comparing wild-type and *ucr-2.3(uth252)* animals fed *cco-1* RNAi in the *rgef-1p::Q40::YFP; hsp-6p::GFP* genetic background. n > 3.
f. Schematic describing genetic changes in all shown mutant alleles of *ucr-2.3*.
g. Fluorescence comparison of intestinal UPR^MT^ signal in wild-type and *ucr-2.3(uth252)* mutant animals over adult aging (Day 1 – 4 of adulthood). n > 3.

Supplementary Fig 2 Supplementary Figure 2: *ucr-2.3* loss of function does not suppress UPR^ER^ or cytosolic HSR cell non-autonomous signaling, Related to Figure 2
a. Fluorescence imaging of intestinal UPR^ER^ signal. **p = 0.0055; n = 3.
b. Fluorescence imaging of intestinal HSR signal. ****p < 0.0001; n = 2.
c. Fluorescence imaging and quantification of intestinal UPR^MT^ signal with and without the *ucr-2.3(uth252)* mutation in the absence of mitochondrial stress (basal condition). p = 0.6179; n = 3.
d. qRT-PCR comparison of *ucr-2.3* expression levels in wildtype and *ucr-2.3(ΔMTS)* mutant animals in the *rgef-1p::Q40::YFP; hsp-6p::GFP* genetic background. Transcript levels normalized by *rpl-32*. p = 0.1603; n = 3.

Supplementary Fig 3 Supplementary Figure 3: *ucr-2.1* and *ucr-2.2* are functionally redundant and display similar tissue expression patterns but differ from *ucr-2.3*, Related to Figure 3
a. Measurement of mitochondrial respiration (OCR) in the *hsp-6p::GFP* genetic background. ****p<0.0001, non-significant p values > 0.4; n = 3.
b. Fluorescence imaging comparison of UPR^MT^ activation. n = 3.
c. Fluorescence imaging comparison of UPR^MT^ activation in the *ucr-2.3(uth252); rgef-1p::Q40::YFP; hsp-6p::GFP* genetic background. n = 3.
d. qRT-PCR measurement of relative transcript levels of the *ucr-2* family genes between the *ucr-2.3(uth252)* loss of function mutant and wildtype in the *rgef-1p::Q40::YFP; hsp-6p::GFP* genetic background. n = 3.
e. qRT-PCR measurement of relative transcript levels of the *ucr-2* family genes between the *ucr-2.1(uth267)* mutant *(i)* and *ucr-2.2(uth263)* mutant *(ii)* and wildtype with RNAi single and double knockdown of *ucr-2.1* and *ucr-2.2 (iii)* in the *rgef-1p::Q40::YFP; hsp-6p::GFP* genetic background. n = 3.
f. Comparison of tissue-expression profiles for the UCR-2 family genes. Plots were generated using the Worm tissue expression prediction web interface^24^ (http://https://worm.princeton.edu/).
g. Fluorescence widefield imaging of *ucr-2.3::GFP* strain in the germline region. Scale bar = 50 μm. Image shown is representative of at least 5–10 animals imaged.

Supplementary Fig 4 Supplementary Figure 4: Additional genetic evidence supporting a germline-specific role for *ucr-2.3* and germline mitochondria in mediating cell non-autonomous UPR^MT^, Related to Figure 4
a. Fluorescence imaging comparison *(i)* and quantification *(ii)* of intestinal UPR^MT^ signal between *ucr-2.3* excised only in neurons using FLP/FRT recombination *ucr-2.3(FRT); rgef-1p::FLP* compared to *ucr-2.3* with the FRT sites alone *ucr-2.3(FRT)*, all in the *rgef-1p::Q40::YFP; hsp-6p::GFP* genetic background. Wild-type and *ucr-2.3(uth252)* loss of function mutant displayed for comparison. **p=0.0052, non-significant p values > 0.4; n = 3. *(iii)* DNA gel displaying excised *ucr-2.3* upon FLP/FRT recombination by PCR genotyping the *ucr-2.3* genetic locus in each strain listed.
b. Measurement of total brood size. ****p < 0.0001; n = 2.
c. *(i)* DIC imaging of oocytes in gonad region. Blue arrowheads point to the oocytes that were quantified in *(ii)*. ****p < 0.0001, n = 2.
d. *(i)* Raw averaged traces of mitochondrial respiration (OCR). The mitochondrial uncoupler FCCP was injected to measure maximum mitochondrial respiration, followed by sodium azide, a complex IV inhibitor, to fully inhibit mitochondrial respiration and measure non-mitochondrial oxygen consumption. *(ii)* Averages of uncoupled respiration during FCCP addition; n = 2.
e. Log_2_(fold change) comparison of changes in gene expression of compiled mitochondrial genes (mean ± SEM = −0.2752 ± 0.04405) and mitochondrial ETC subunit genes (mean ± SEM = −0.2356 ± 0.1321) between wild-type and *ucr-2.3(uth252)* RNA-seq datasets. For a list of considered mitochondrial genes, see Supplementary Table 1.
f. Comparison of tissue-expression profiles for the *nduf-2* family genes (*gas-1/nduf-2.1*, *nduf-2.2*). Plots were generated using the Worm tissue expression prediction web interface^24^ (https://worm.princeton.edu/).
g. Fluorescence imaging (*i)* and quantification *(ii)* of intestinal UPR^MT^ signal. p-value = 0.5335; n = 2.
h. *(i)* Timeline schematic of mtKillerRed activation experiment. Animals were exposed to green light at the Day 1 (D1) developmental stage to activate mtKillerRed after development and expansion of the germline in the L3-L4 larval stages. Fluorescence imaging *(ii)* and quantification *(iii)* of intestinal UPR^MT^ signal between wild-type and *pie-1::mtKillerRed* animals was assessed at Day 2 (D2), as done in a similar protocol^36^. Non-significant p-value = 0.2750, **p = 0.0022; n = 2.

Supplementary Fig 5 Supplementary Figure 5: Additional evidence supporting a specific role for the germline in mediating UPR^MT^ signaling, Related to Figure 5
a. Fluorescence widefield imaging of *pie-1p::tomm-20::mKate2* germline mitochondrial reporter strain with and without FUDR treatment.
b. Fluorescence imaging comparison and quantification of the cell non-autonomous UPR^ER^ *(i)* and cytosolic HSR *(ii)* reporter strains upon genetic germline depletion by the *glp-4(bn2)* temperature sensitive mutation at the restrictive temperature 25°C. Non-significant p = 0.86, n = 3; ***p = 0.0005, n = 4.

Supplementary Fig 6 Supplementary Figure 6: Germline-specific knockdown of known mitokine receptors and UPR^MT^ regulators does not suppress non-autonomous UPR^MT^ signaling, Related to Figure 6
a. Schematic of a possible “Direct Signaling” model, in which stressed neurons release mitokine signals that are directly received by the germline. The germline processes these neuronal mitokine signals and, in turn, sends its own signal to the intestine to regulate UPR^MT^ activation.
b. Fluorescence imaging comparison *(i)* and quantification *(ii)* of intestinal UPR^MT^ signal for RNAi knockdown of Frizzled receptors in a germline-specific RNAi strain^64^ crossed to the cell non-autonomous UPR^MT^ reporter: *sun-1p::rde-1; rde-1(mkc36); rgef-1p::Q40::YFP; hsp-6p::GFP*. *p = 0.0357, non-significant p-values > 0.4. n = 2.
c. Fluorescence imaging comparison *(i)* and quantification *(ii)* of intestinal UPR^MT^ signal for RNAi knockdown of serotonin receptors in the germline-specific RNAi strain crossed to the cell non-autonomous UPR^MT^ reporter. Non-significant p-values > 0.12. n = 2.
d. Fluorescence imaging comparison *(i)* and quantification *(ii)* of intestinal UPR^MT^ signal for RNAi knockdown of UPR^MT^ factors in the germline-specific RNAi strain crossed to the cell non-autonomous UPR^MT^ reporter. Non-significant p-values > 0.22. n = 3.

Supplementary Fig 7 Supplementary Figure 7: Germline deficient and *ucr-2.3* mutant animals have increased intestinal fat, Related to Figure 7
a. Oil Red O staining *(i)* and quantification *(ii)* of lipid levels. **p = 0.0185; n = 2.
b. Oil Red O staining *(i)* and quantification *(ii)* of lipid levels in the *rgef-1p::Q40::YFP; hsp-6p::GFP* genetic background. ***p = 0.0002; n = 2.
c. Nile red staining *(i)* and quantification *(ii)* of intestinal lipid levels. Non-significant p = 0.3958, *p = 0.0334, ****p < 0.0001; n=2.
d. Pumping rate of *ucr-2.3* loss of function mutants animals in the wild-type and *rgef-1p::Q40::YFP; hsp-6p::GFP* genetic backgrounds. *p = 0.0350; n= 2.
e. Comparison of log_2_(fold change) of expression changes in fatty acid synthesis, metabolism, and transport genes from RNA-seq datasets of the *ucr-2.3(uth252); rgef-1p::Q40::YFP* mutant compared to *rgef-1p::Q40::YFP* alone.
f. Nile red staining *(i)* and quantification *(ii)* of intestinal lipid levels conducted at the restrictive temperature 25°C. ****p <0.0001; n = 2.
g. Nile red staining *(i)* and quantification *(ii)* of intestinal lipid levels. *p = 0.038; n=2.

Supplementary Table 1 Supplemental Table 1: Differentially regulated mitochondrial and mitochondrial ETC transcripts from the *ucr-2.3(uth252)* mutation, Related to Figure 4
Excel file list of differentially regulated mitochondrial and mitochondrial ETC genes considered in Supplementary Figure 4E from RNA-seq dataset compared *ucr-2.3(uth252); rgef-1p::Q40::YFP* animals with wild-type *rgef-1p::Q40::YFP* animals. Log_2_(fold change) values and adjusted p-value also listed.

## Figures and Tables

**KEY RESOURCES TABLE:** 

REAGENT or RESOURCE	SOURCE	IDENTIFIER
**Antibodies**		
Anti-HA tag antibody	Abcam	Cat#ab9110; RRID:AB_307019
Anti-NDUFS3 antibody [17D95]	Abcam	Cat#ab14711; RRID:AB_301429
Anti-α-Tubulin antibody	Sigma-Aldrich	Cat#T9026; RRID:AB_477593
**Bacterial and Virus Strains**		
OP50	CGC	OP50, RRID:WB-STRAIN:WBStrain00041969
HT115	CGC	HT115, RRID:WB-STRAIN:WBStrain00041079
Stbl3	Thermo-Fisher	C7373-03
**Chemicals, Peptides, and Recombinant Proteins**		
Cas9 recombinant protein	QB3 MacroLab (University of California, Berkeley)	N/A
FCCP	Sigma-Aldrich	C2920
Sodium azide	Sigma-Aldrich	S2002
FUDR	Spectrum Chemical	50-91-9
Ethyl methanesulfonate	Sigma-Aldrich	M0880
Serotonin-HCl	Sigma-Aldrich	H9523
Critical Commercial Assays		
Seahorse XFe96 FluxPak	Agilent Technologies	102416-100
Deposited Data		
RNA-seq	Mendeley	10.17632/hpbddm6k5p.2
**Experimental Models: Cell Lines**		
Human hTERT-RPE-1 PAC knockout cells	Andrew Holland	Plk4^AS^ hTERT-RPE1
HEK293T	ATCC	CRL-11268, RRID:CVCL_1926
**Experimental Models: Organisms/Strains**		
*C. elegans*: Bristol (N2) strain as wild type (WT)	CGC	N2, RRID:WB-STRAIN:WBStrain00000001
*C. elegans*: AGD3126: *zcIs13[hsp-6p::GFP] V*(SJ4100 backcrossed 6X)	CGC	SJ4100, RRID:WB-STRAIN:WBStrain00034068
*C. elegans*: AGD785: *rmIs110[rgef-1p::Q40::YFP];zcIs13 V[hsp-6p::GFP]*	Berendzen and Durieux et al., Cell 2016^12^	AGD785
*C. elegans*: AGD2987: *ucr-2.3(uth252); rmIs110[rgef-1p::Q40::YFP];zcIs13 V[hsp-6p::GFP]*	This study	AGD2987
*C. elegans*: AGD3547: *ucr-2.3(uth252); sid-1(qt9); uthIs243[rab-3p::cco-1HP, myo-2p::tdTomato]; zcIs13[hsp-6p::GFP]*	This study	AGD3547
*C. elegans*: AGD1072: *sid-1(qt9); uthIs243[rab-3p::cco-1HP, myo-2p::tdTomato]; zcIs13[hsp-6p::GFP]*	This study	AGD1072
*C. elegans*: PHX6175: *ucr-2.3(syb6175) (3x HA tag)*	This study (SunyBiotech)	PHX6175
*C. elegans*: AGD3264: *ucr-2.3(uth252)*	This study	AGD3264
*C. elegans*: AGD3606: *ucr-2.3(syb6228); rmIs110[rgef-1p::Q40::YFP];zcIs13 V[hsp-6p::GFP]* (ΔMTS mutant)	This study	AGD3606
*C. elegans*: PHX4838: *ucr-2.1(syb4838) [ucr-2.1p::mCherry::GSG::F2A::ucr-2.1]*	This study (SunyBiotech)	PHX4838
*C. elegans*: PHX2912: *ucr-2.2(syb2912) [ucr-2.2p::mCherry::GSG::F2A::ucr-2.2]*	This study (SunyBiotech)	PHX2912
*C. elegans*: PHX3476: *ucr-2.3(syb3476) [ucr-2.3p::mCherry::GSG::F2A::ucr-2.3]*	This study (SunyBiotech)	PHX3476
*C. elegans*: NW1229: *evIs111[F25B3.3::GFP + dpy-20(*+*)]*	CGC	NW1229; RRID:WB-STRAIN:WBStrain00029126
*C. elegans*: AGD3550: *ucr-2.3(syb3476); evIs111[F25B3.3::GFP + dpy-20(*+*)]*	This study	AGD3550
*C. elegans*: AGD3131: *ucr-2.1(uth267); rmIs110[rgef-1p::Q40::YFP];zcIs13 V[hsp-6p::GFP]*	This study	AGD3131
*C. elegans*: AGD3020: *ucr-2.2(uth263); rmIs110[rgef-1p::Q40::YFP];zcIs13 V[hsp-6p::GFP]*	This study	AGD3020
*C. elegans*: EG6703: oxEx1582 [eft-3p::GFP + Cbr-unc-119(+)]	CGC	EG6703; RRID:WB-STRAIN:WBStrain00006740
*C. elegans*: AGD3676: *ucr-2.3(ok3073); uthSi115[rgef-1p::ucr-2.3 gDNA::tbb-2 3′UTR]; rmIs110[rgef-1p::Q40::YFP]; zcIs13 V[hsp-6p::GFP]*	This study	AGD3676
*C. elegans*: AGD3679: *ucr-2.3(ok3073); uthSi116[pie-1p::ucr-2.3 gDNA::tbb-2 3′UTR]; rmIs110[rgef-1p::Q40::YFP]; zcIs13 V[hsp-6p::GFP]*	This study	AGD3679
*C. elegans*: VC2360: *ucr-2.3(ok3073)*	CGC	VC2360; RRID:WB-STRAIN:WBStrain00037284
*C. elegans*: AGD3324: *ucr-2.3(ok3073); rmIs110[rgef-1p::Q40::YFP]; zcIs13 V[hsp-6p::GFP]*	This study	AGD3324
*C. elegans*: SS104: *glp-4(bn2)*	CGC	SS104; RRID:WB-STRAIN:WBStrain00034434
*C. elegans*: AGD3528: *ucr-2.3(uth252); glp-4(bn2); rmIs110[rgef-1p::Q40::YFP]; zcIs13 V[hsp-6p::GFP]*	This study	AGD3528
*C. elegans*: AGD3605: *nduf-2.2(uth312); rmIs110[rgef-1p::Q40::YFP]; zcIs13 V[hsp-6p::GFP]*	This study	AGD3605
*C. elegans*: SJZ106: *foxSi27[pie-1p::tomm20::mKate2::HA:: tbb-2 3′UTR]*	Dr. Steven Zuryn (University of Queensland)	SJZ106
*C. elegans*: AGD3684: *ucr-2.3(uth252); foxSi27[pie-1p::tomm20::mKate2::HA:: tbb-2 3′UTR]*	This study	AGD3684
*C. elegans*: AGD3802: *ucr-2.3(ok3073); rmIs110[rgef-1p::Q40::YFP]; zcIs13 V[hsp-6p::GFP]; uthSi119[pie-1p::ucr-2.1:: tbb-2 3′UTR]*	This study	AGD3802
*C. elegans*: CB4037: *glp-1(e2141)*	CGC	CB4037; RRID:WB-STRAIN:WBStrain00004531
*C. elegans*: AGD3548: *glp-1(e2141); rmIs110[rgef-1p::Q40::YFP]; zcIs13 V[hsp-6p::GFP]*	This study	AGD3548
*C. elegans*: AGD3407: *glp-4(bn2); rmIs110[rgef-1p::Q40::YFP]; zcIs13 V[hsp-6p::GFP]*	This study	AGD3407
*C. elegans*: AGD3689: *glp-4(bn2); sid-1(qt9); uthls243[rab-3p::cco-1HP, myo-2p::tdTomato]; zclsl 3[hsp-6p::GFP]*	This study	AGD3689
*C. elegans*: AGD3207: *uthIs409[myo-2p::tdtomato, rgef-1p::jmjd-1.2a::3’UTR unc-54], zcIs13[hsp-6p::GFP]*	This study	AGD3207
*C. elegans*: AGD3386: *ucr-2.3(uth252); uthIs409[myo-2p::tdtomato, rgef-1p::jmjd-1.2a::3′UTR unc-54], zcIs13[hsp-6p::GFP]*	This study	AGD3386
*C. elegans*: LTY43: *ythIs3[rgef-1p::egl-20, myo-2p::tdTomato]; zcIs13[hsp-6p::GFP]*	Zhang et al., Cell 2018^16^	LTY43
*C. elegans*: AGD3325: *ucr-2.3(uth252); ythIs3[rgef-1p::egl-20, myo-2p::tdTomato]; zcIs13[hsp-6p::GFP]*	This study	AGD3325
*C. elegans*: AGD3263: *rgef-1p::Q40::YFP* (backcrossed AM101)	CGC	AM101; RRID:WB-STRAIN:WBStrain00000178
*C. elegans*: AGD1415: *pwIs23[vit-2::GFP]* (backcrossed RT130)	CGC	RT130; RRID:WB-STRAIN:WBStrain00033468
*C. elegans*: AGD3870: *ucr-2.3(uth252); pwIs23[vit-2::GFP]* (backcrossed RT130)	This study	AGD3870
*C. elegans*: AGD2866: *ucr-2.3(uth216); rmIs110[rgef-1p::Q40::YFP]; zcIs13 V[hsp-6p::GFP]*	This study	AGD2866
*C. elegans*: AGD2864: *ucr-2.3(uth214); rmIs110[rgef-1p::Q40::YFP]; zcIs13 V[hsp-6p::GFP]*	This study	AGD2864
*C. elegans*: AGD1908: *N2, uthIs464[rgef-1p::xbp-1s, myo-2p::tdTomato]; zcls4[hsp-4p::GFP]V*	This study	AGD1908
*C. elegans*: AGD3691: *glp-4(bn2), uthIs464[rgef-1p::xbp-1s, myo-2p::tdTomato]; zcls4[hsp-4p::GFP]V*	This study	AGD3691
*C. elegans*: AGD1448: *dvln70[pCL25 (hsp-16.2p::GFP), pRF4(rol-6)]; uthIS368[rab-3p::hsf-1 FL, myo-2p::tdTomato]*	Baird and Douglas et al., Science 2014^73^	AGD1448
*C. elegans*: AGD3690: *glp-4(bn2); dvln70[pCL25 (hsp-16.2p::GFP), pRF4(rol-6)]; uthIS368[rab-3p::hsf-1 FL, myo-2p::tdTomato]*	This study	AGD3690
*C. elegans*: AGD3134: *ucr-2.3(uth252); zcIs13 V[hsp-6p::GFP]*	This study	AGD3134
*C. elegans*: APW202: *ucr-2.3::GFP*	Dr. Andrew Wojtovich (University of Rochester Medical Center)	APW202
*C. elegans*: AGD3620: *ucr-2.3(syb6154 syb6260); rgef-1p::Q40::YFP; hsp-6p::GFP*	This study	AGD3620
*C. elegans*: AGD3657: *ucr-2.3(syb6154 syb6260); rgef-1p::FLP; rmIs110[rgef-1p::Q40::YFP]; zcIs13 V[hsp-6p::GFP]*	This study	AGD3657
*C. elegans*: AGD3655: *mkcSi13 [sun-1p::rde-1::sun-1 3′UTR + unc-119(+)], rgef-1p::Q40::YFP; hsp-6p::GFP*	This study, mkcSi13 [sun-1p::rde-1::sun-1 3′UTR + unc-119(+)]	AGD3655
	from Zou et al., Sci Rep 2019^64^	
C. elegans: AGD3898; *pie-1p::mtKillerRed; rmIs110[rgef-1p::Q40::YFP]; zcIs13 V[hsp-6p::GFP]*	This study	AGD3898
**Oligonucleotides**		
rpl-32 FWD, AGGGAATTGATAACCGTGTCCGCA	Ramachandran et al., Dev Cell 2019^74^	N/A
rpl-32 REV, TGTAGGACTGCATGAGGAGCATGT	Ramachandran et al., Dev Cell 2019^74^	N/A
hsp-6 FWD, AGC CAAGTTCGAGCAGATTG	Ramachandran et al., Dev Cell 2019^74^	N/A
hsp-6 REV, TTGCACCTTTGGCATTCTGC	Ramachandran et al., Dev Cell 2019^74^	N/A
ucr-2.1 FWD, TTGAACTAAACGGAGCCAC	This study	N/A
ucr-2.1 REV, TAAGTCCTTGCTTGTTGGC	This study	N/A
ucr-2.2 FWD, CGAGGATGTGCTGCCAACTA	This study	N/A
ucr-2.2 REV, CGAAGTGCTGCTCTGCAAAT	This study	N/A
ucr-2.3 FWD, GGAGTGTGGTGTCGTACCAG	This study	N/A
ucr-2.3 REV, GCGATAGTCTCCACCCCTGA	This study	N/A
**Recombinant DNA**		
UCR-2.3-mNeonGreen (CD510-B1 plasmid backbone)	This study	N/A
OMP25-tagBFP2 (CD510-B1 plasmid backbone)	This study	N/A
pie-1p::ucr-2.3_gDNA::tbb-2_3′UTR (pCFJ356 plasmid backbone)	This study	N/A
pie-1p::ucr-2.1_gDNA::tbb-2_3′UTR (pCFJ356 plasmid backbone)	This study	N/A
rgef-1p::ucr-2.3_gDNA::tbb-2_3′UTR (pCFJ356 plasmid backbone)	This study	N/A
pie-1p::mtKillerRed::tbb-2_3′UTR (pCFJ356 plasmid backbone)	This study	N/A
**Software and Algorithms**		
Prism 10	GraphPad (commercially available)	https://www.graphpad.com
Image Studio Lite	LI-COR (commercially available)	https://www.licor.com
FIJI	FIJI (commercially available)	https://fiti.sc
