# Supplementary figures and images for: The germline coordinates mitokine signaling
Source: Cell. Author manuscript; Available in PMC 2025 Jul 15. (PMC12261959; doi:10.1016/j.cell.2024.06.010)

# Supplementary Figure 1

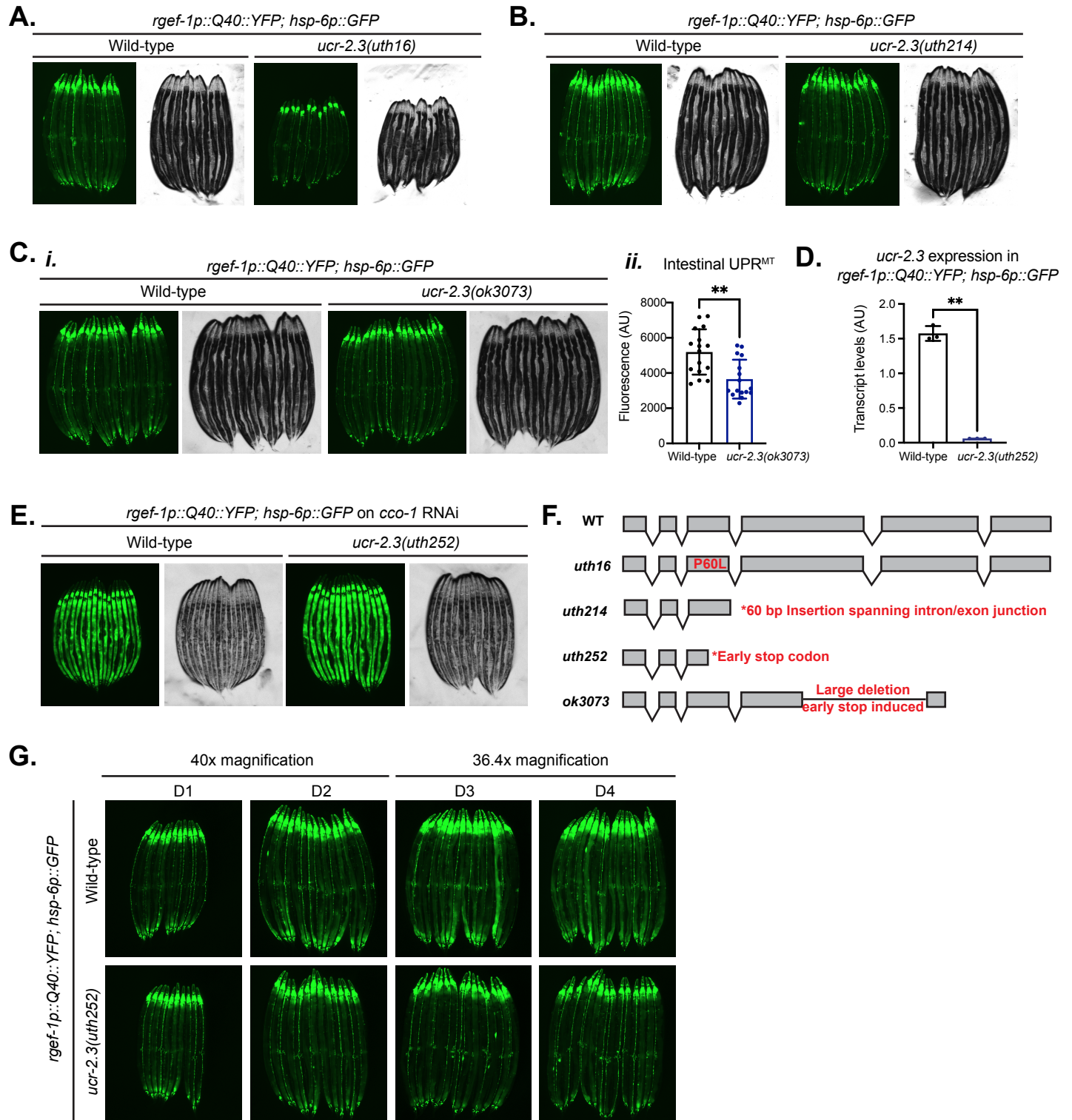

Supplement: Supplementary Fig 1 — Supplementary Figure 1: Additional genetic evidence supporting loss of function mutations in ucr-2.3 suppress cell non-autonomous UPRMT signaling in C. elegans, Related to Figure 1 a. Fluorescence comparison of intestinal UPRMT signal in the rgef-1p::Q40::YFP;hsp-6p::GFP genetic background with and without the EMS mutant allele obtained in the suppressor screen uth16. n > 3. b. Fluorescence comparison of intestinal UPRMT signal with and without a loss of function mutation ucr-2.3(uth214) generated by CRISPR/Cas9 genome editing. n > 3. c. Fluorescence imaging (i) and quantification (ii) of intestinal UPRMT signal with and without a large deletion mutation ucr-2.3(ok3073). p = 0.0010; n > 3. d. qRT-PCR comparison of ucr-2.3 expression levels in wild-type and ucr-2.3(uth252) mutant animals in the rgef-1p::Q40::YFP; hsp-6p::GFP genetic background. Transcript levels normalized by rpl-32. **p=0.0016; n = 3. e. Fluorescence imaging showing autonomous UPRMT activation comparing wild-type and ucr-2.3(uth252) animals fed cco-1 RNAi in the rgef-1p::Q40::YFP; hsp-6p::GFP genetic background. n > 3. f. Schematic describing genetic changes in all shown mutant alleles of ucr-2.3. g. Fluorescence comparison of intestinal UPRMT signal in wild-type and ucr-2.3(uth252) mutant animals over adult aging (Day 1 – 4 of adulthood). n > 3. [file NIHMS2092584-supplement-Supplementary_Fig_1.pdf]

## Supplementary Figure 2

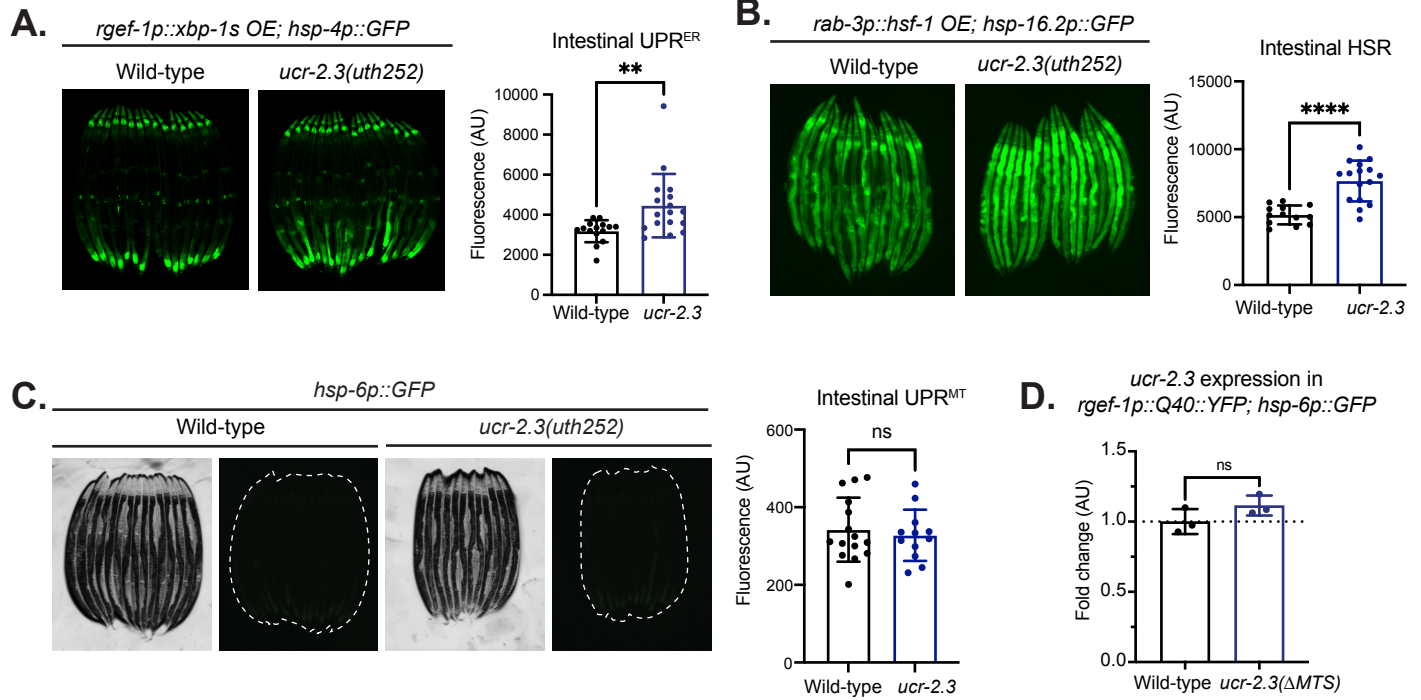

Supplement: Supplementary Fig 2 — Supplementary Figure 2: ucr-2.3 loss of function does not suppress UPRER or cytosolic HSR cell non-autonomous signaling, Related to Figure 2 a. Fluorescence imaging of intestinal UPRER signal. **p = 0.0055; n = 3. b. Fluorescence imaging of intestinal HSR signal. ****p < 0.0001; n = 2. c. Fluorescence imaging and quantification of intestinal UPRMT signal with and without the ucr-2.3(uth252) mutation in the absence of mitochondrial stress (basal condition). p = 0.6179; n = 3. d. qRT-PCR comparison of ucr-2.3 expression levels in wildtype and ucr-2.3(ΔMTS) mutant animals in the rgef-1p::Q40::YFP; hsp-6p::GFP genetic background. Transcript levels normalized by rpl-32. p = 0.1603; n = 3. [file NIHMS2092584-supplement-Supplementary_Fig_2.pdf]

# Supplementary Figure 3

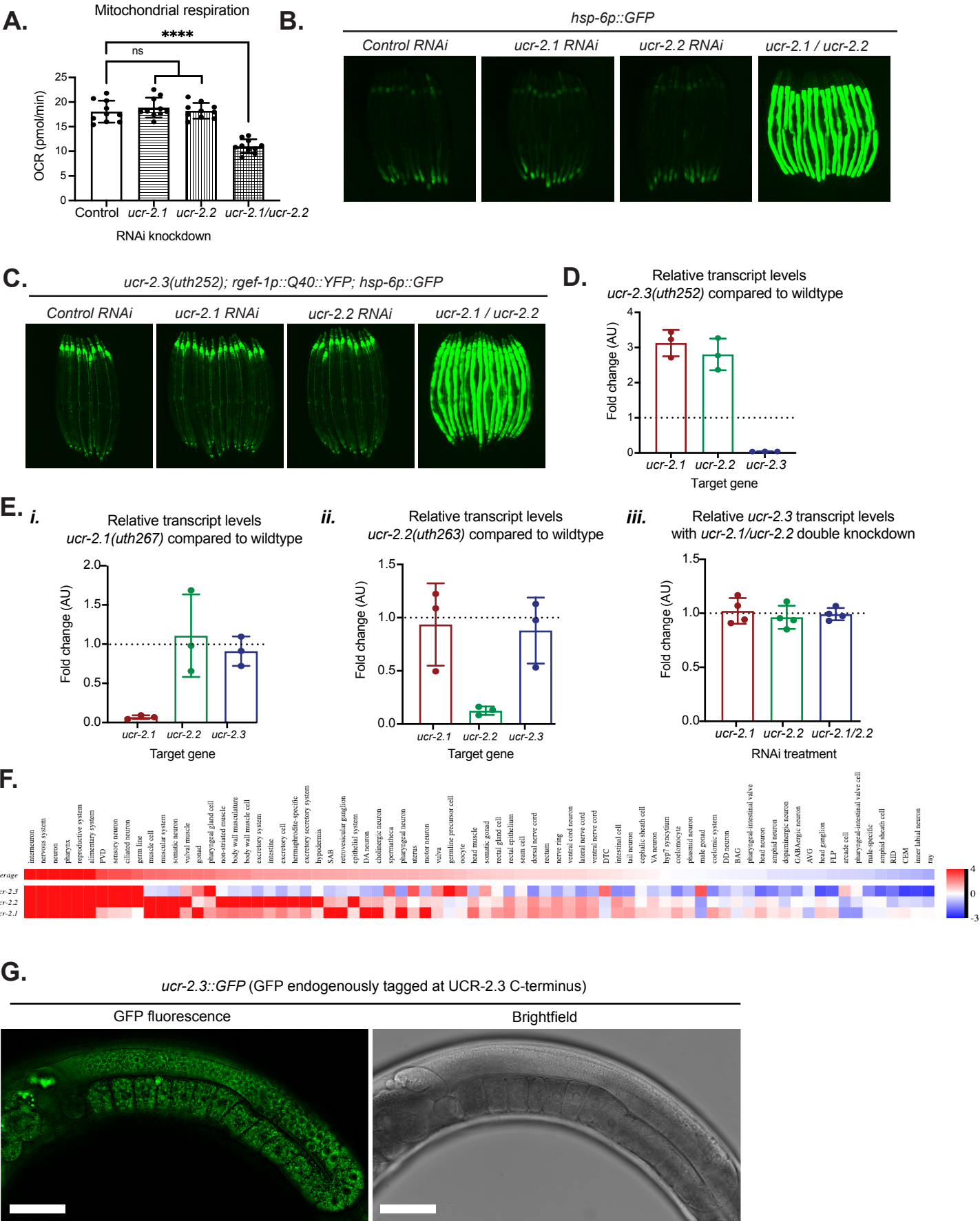

Supplement: Supplementary Fig 3 — Supplementary Figure 3: ucr-2.1 and ucr-2.2 are functionally redundant and display similar tissue expression patterns but differ from ucr-2.3, Related to Figure 3 a. Measurement of mitochondrial respiration (OCR) in the hsp-6p::GFP genetic background. ****p<0.0001, non-significant p values > 0.4; n = 3. b. Fluorescence imaging comparison of UPRMT activation. n = 3. c. Fluorescence imaging comparison of UPRMT activation in the ucr-2.3(uth252); rgef-1p::Q40::YFP; hsp-6p::GFP genetic background. n = 3. d. qRT-PCR measurement of relative transcript levels of the ucr-2 family genes between the ucr-2.3(uth252) loss of function mutant and wildtype in the rgef-1p::Q40::YFP; hsp-6p::GFP genetic background. n = 3. e. qRT-PCR measurement of relative transcript levels of the ucr-2 family genes between the ucr-2.1(uth267) mutant (i) and ucr-2.2(uth263) mutant (ii) and wildtype with RNAi single and double knockdown of ucr-2.1 and ucr-2.2 (iii) in the rgef-1p::Q40::YFP; hsp-6p::GFP genetic background. n = 3. f. Comparison of tissue-expression profiles for the UCR-2 family genes. Plots were generated using the Worm tissue expression prediction web interface24 (http://https://worm.princeton.edu/). g. Fluorescence widefield imaging of ucr-2.3::GFP strain in the germline region. Scale bar = 50 μm. Image shown is representative of at least 5–10 animals imaged. [file NIHMS2092584-supplement-Supplementary_Fig_3.pdf]

# Supplementary Figure 4

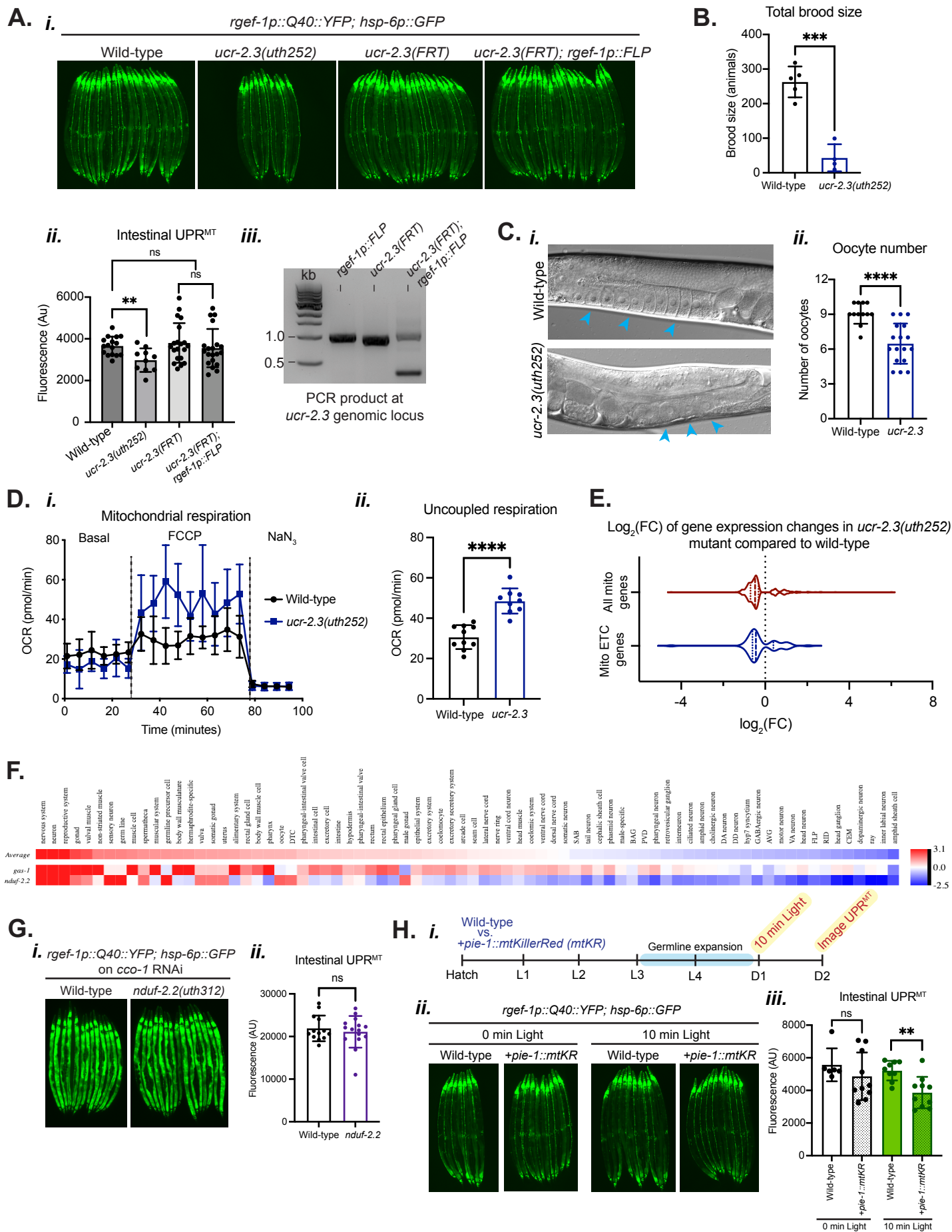

Supplement: Supplementary Fig 4 — Supplementary Figure 4: Additional genetic evidence supporting a germline-specific role for ucr-2.3 and germline mitochondria in mediating cell non-autonomous UPRMT, Related to Figure 4 a. Fluorescence imaging comparison (i) and quantification (ii) of intestinal UPRMT signal between ucr-2.3 excised only in neurons using FLP/FRT recombination ucr-2.3(FRT); rgef-1p::FLP compared to ucr-2.3 with the FRT sites alone ucr-2.3(FRT), all in the rgef-1p::Q40::YFP; hsp-6p::GFP genetic background. Wild-type and ucr-2.3(uth252) loss of function mutant displayed for comparison. **p=0.0052, non-significant p values > 0.4; n = 3. (iii) DNA gel displaying excised ucr-2.3 upon FLP/FRT recombination by PCR genotyping the ucr-2.3 genetic locus in each strain listed. b. Measurement of total brood size. ****p < 0.0001; n = 2. c. (i) DIC imaging of oocytes in gonad region. Blue arrowheads point to the oocytes that were quantified in (ii). ****p < 0.0001, n = 2. d. (i) Raw averaged traces of mitochondrial respiration (OCR). The mitochondrial uncoupler FCCP was injected to measure maximum mitochondrial respiration, followed by sodium azide, a complex IV inhibitor, to fully inhibit mitochondrial respiration and measure non-mitochondrial oxygen consumption. (ii) Averages of uncoupled respiration during FCCP addition; n = 2. e. Log2(fold change) comparison of changes in gene expression of compiled mitochondrial genes (mean ± SEM = −0.2752 ± 0.04405) and mitochondrial ETC subunit genes (mean ± SEM = −0.2356 ± 0.1321) between wild-type and ucr-2.3(uth252) RNA-seq datasets. For a list of considered mitochondrial genes, see Supplementary Table 1. f. Comparison of tissue-expression profiles for the nduf-2 family genes (gas-1/nduf-2.1, nduf-2.2). Plots were generated using the Worm tissue expression prediction web interface24 (https://worm.princeton.edu/). g. Fluorescence imaging (i) and quantification (ii) of intestinal UPRMT signal. p-value = 0.5335; n = 2. h. (i) Timeline schematic of mtKillerRe [file NIHMS2092584-supplement-Supplementary_Fig_4.pdf]

## Supplementary Figure 5

**A.**

*pie-1p::tomm-20::mKate2*

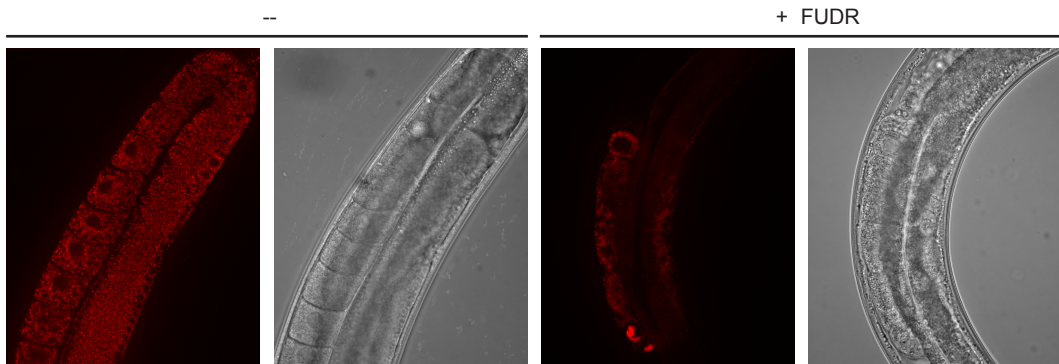

**B. i.** *rgef-1p::xbp-1s OE; hsp-4p::GFP*

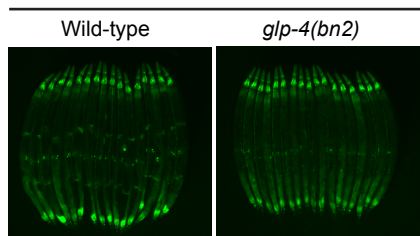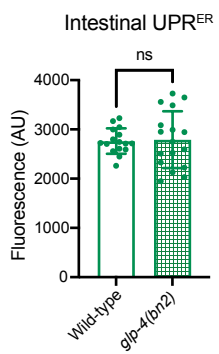

**ii.** *rab-3p::hsf-1s* OE; *hsp-16.2p::GFP*

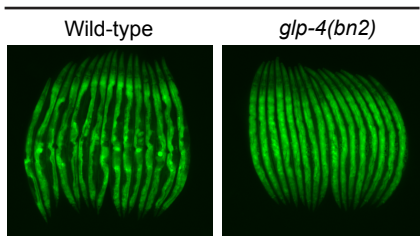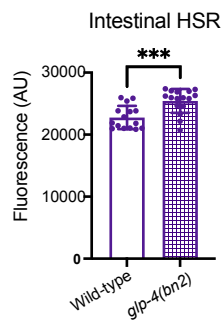

Supplement: Supplementary Fig 5 — Supplementary Figure 5: Additional evidence supporting a specific role for the germline in mediating UPRMT signaling, Related to Figure 5 a. Fluorescence widefield imaging of pie-1p::tomm-20::mKate2 germline mitochondrial reporter strain with and without FUDR treatment. b. Fluorescence imaging comparison and quantification of the cell non-autonomous UPRER (i) and cytosolic HSR (ii) reporter strains upon genetic germline depletion by the glp-4(bn2) temperature sensitive mutation at the restrictive temperature 25°C. Non-significant p = 0.86, n = 3; ***p = 0.0005, n = 4. [file NIHMS2092584-supplement-Supplementary_Fig_5.pdf]

Supplementary Figure 6

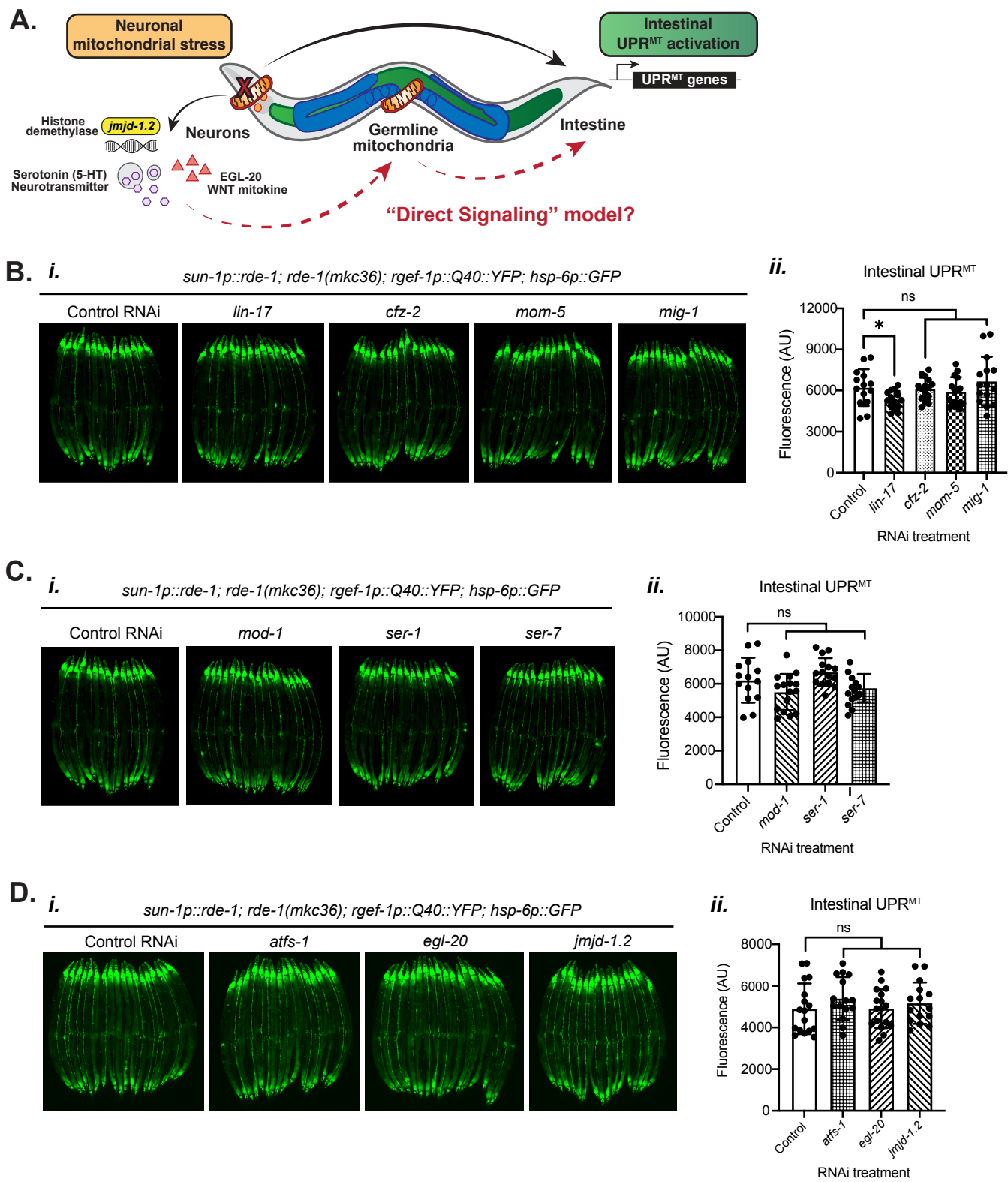

Supplement: Supplementary Fig 6 — Supplementary Figure 6: Germline-specific knockdown of known mitokine receptors and UPRMT regulators does not suppress non-autonomous UPRMT signaling, Related to Figure 6 a. Schematic of a possible “Direct Signaling” model, in which stressed neurons release mitokine signals that are directly received by the germline. The germline processes these neuronal mitokine signals and, in turn, sends its own signal to the intestine to regulate UPRMT activation. b. Fluorescence imaging comparison (i) and quantification (ii) of intestinal UPRMT signal for RNAi knockdown of Frizzled receptors in a germline-specific RNAi strain64 crossed to the cell non-autonomous UPRMT reporter: sun-1p::rde-1; rde-1(mkc36); rgef-1p::Q40::YFP; hsp-6p::GFP. *p = 0.0357, non-significant p-values > 0.4. n = 2. c. Fluorescence imaging comparison (i) and quantification (ii) of intestinal UPRMT signal for RNAi knockdown of serotonin receptors in the germline-specific RNAi strain crossed to the cell non-autonomous UPRMT reporter. Non-significant p-values > 0.12. n = 2. d. Fluorescence imaging comparison (i) and quantification (ii) of intestinal UPRMT signal for RNAi knockdown of UPRMT factors in the germline-specific RNAi strain crossed to the cell non-autonomous UPRMT reporter. Non-significant p-values > 0.22. n = 3. [file NIHMS2092584-supplement-Supplementary_Fig_6.pdf]

Supplemental Figure 7

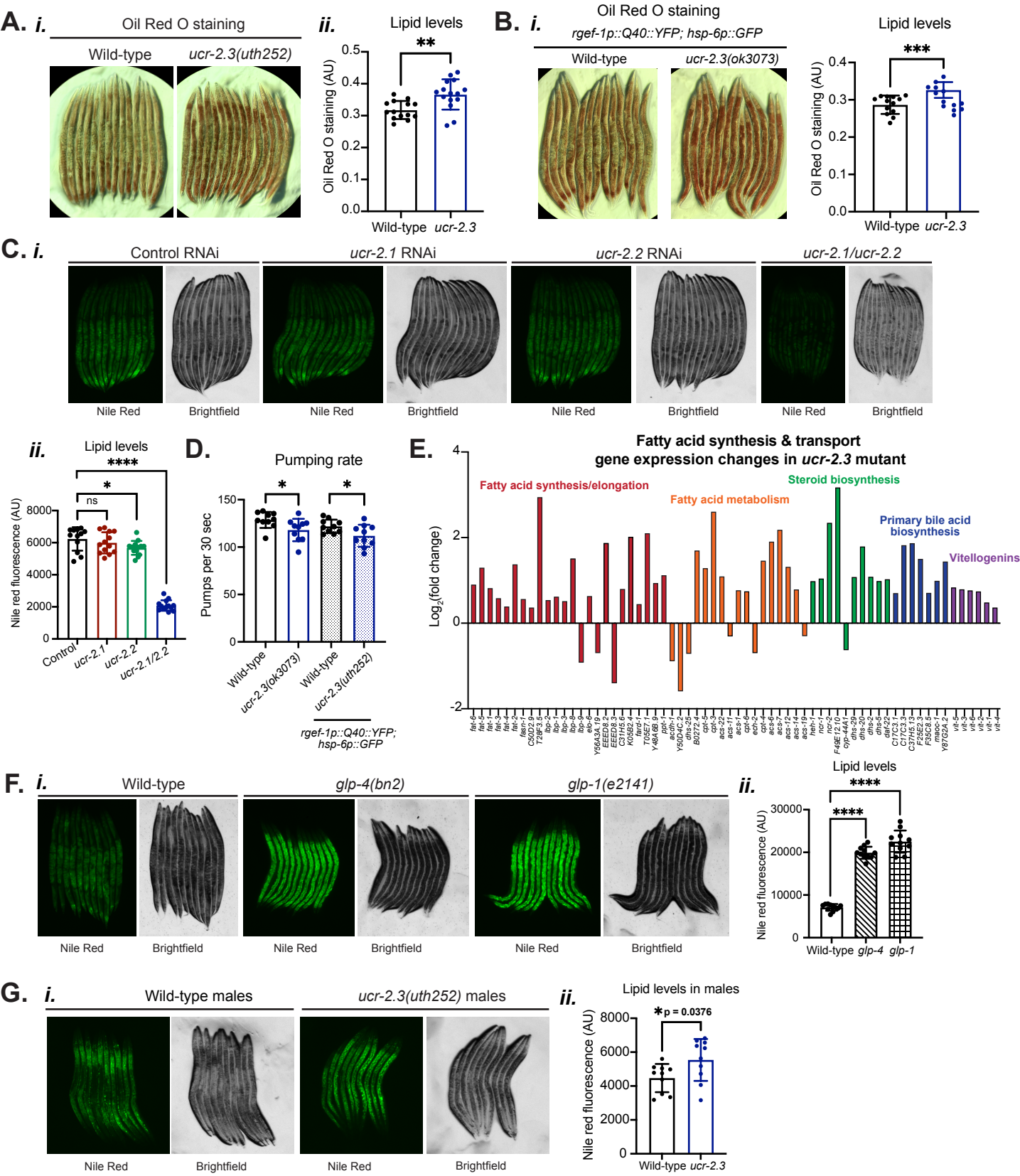

Supplement: Supplementary Fig 7 — Supplementary Figure 7: Germline deficient and ucr-2.3 mutant animals have increased intestinal fat, Related to Figure 7 a. Oil Red O staining (i) and quantification (ii) of lipid levels. **p = 0.0185; n = 2. b. Oil Red O staining (i) and quantification (ii) of lipid levels in the rgef-1p::Q40::YFP; hsp-6p::GFP genetic background. ***p = 0.0002; n = 2. c. Nile red staining (i) and quantification (ii) of intestinal lipid levels. Non-significant p = 0.3958, *p = 0.0334, ****p < 0.0001; n=2. d. Pumping rate of ucr-2.3 loss of function mutants animals in the wild-type and rgef-1p::Q40::YFP; hsp-6p::GFP genetic backgrounds. *p = 0.0350; n= 2. e. Comparison of log2(fold change) of expression changes in fatty acid synthesis, metabolism, and transport genes from RNA-seq datasets of the ucr-2.3(uth252); rgef-1p::Q40::YFP mutant compared to rgef-1p::Q40::YFP alone. f. Nile red staining (i) and quantification (ii) of intestinal lipid levels conducted at the restrictive temperature 25°C. ****p <0.0001; n = 2. g. Nile red staining (i) and quantification (ii) of intestinal lipid levels. *p = 0.038; n=2. [file NIHMS2092584-supplement-Supplementary_Fig_7.pdf]
